# Optimal medical care and coronary flow capacity-guided myocardial revascularization vs usual care for chronic coronary artery disease: the CENTURY trial

**DOI:** 10.1093/eurheartj/ehaf356

**Published:** 2025-05-29

**Authors:** K Lance Gould, Nils P Johnson, Amanda E Roby, Richard Kirkeeide, Mary Haynie, Tung Nguyen, Linh Bui, Monica B Patel, Danai Kitkungvan, Patricia Mendoza, Dejian Lai, Ruosha Li, Stefano Sdringola, David McPherson, Jagat Narula

**Affiliations:** Weatherhead PET Center for Preventing and Reversing Atherosclerosis, Division of Cardiology, Department of Medicine, University of Texas McGovern Medical School, 6431 Fannin St., Room MSB 4.256, and Memorial Hermann Hospital, Houston, TX 77030, USA; Weatherhead PET Center for Preventing and Reversing Atherosclerosis, Division of Cardiology, Department of Medicine, University of Texas McGovern Medical School, 6431 Fannin St., Room MSB 4.256, and Memorial Hermann Hospital, Houston, TX 77030, USA; Weatherhead PET Center for Preventing and Reversing Atherosclerosis, Division of Cardiology, Department of Medicine, University of Texas McGovern Medical School, 6431 Fannin St., Room MSB 4.256, and Memorial Hermann Hospital, Houston, TX 77030, USA; Weatherhead PET Center for Preventing and Reversing Atherosclerosis, Division of Cardiology, Department of Medicine, University of Texas McGovern Medical School, 6431 Fannin St., Room MSB 4.256, and Memorial Hermann Hospital, Houston, TX 77030, USA; Weatherhead PET Center for Preventing and Reversing Atherosclerosis, Division of Cardiology, Department of Medicine, University of Texas McGovern Medical School, 6431 Fannin St., Room MSB 4.256, and Memorial Hermann Hospital, Houston, TX 77030, USA; Weatherhead PET Center for Preventing and Reversing Atherosclerosis, Division of Cardiology, Department of Medicine, University of Texas McGovern Medical School, 6431 Fannin St., Room MSB 4.256, and Memorial Hermann Hospital, Houston, TX 77030, USA; Weatherhead PET Center for Preventing and Reversing Atherosclerosis, Division of Cardiology, Department of Medicine, University of Texas McGovern Medical School, 6431 Fannin St., Room MSB 4.256, and Memorial Hermann Hospital, Houston, TX 77030, USA; Weatherhead PET Center for Preventing and Reversing Atherosclerosis, Division of Cardiology, Department of Medicine, University of Texas McGovern Medical School, 6431 Fannin St., Room MSB 4.256, and Memorial Hermann Hospital, Houston, TX 77030, USA; Weatherhead PET Center for Preventing and Reversing Atherosclerosis, Division of Cardiology, Department of Medicine, University of Texas McGovern Medical School, 6431 Fannin St., Room MSB 4.256, and Memorial Hermann Hospital, Houston, TX 77030, USA; Weatherhead PET Center for Preventing and Reversing Atherosclerosis, Division of Cardiology, Department of Medicine, University of Texas McGovern Medical School, 6431 Fannin St., Room MSB 4.256, and Memorial Hermann Hospital, Houston, TX 77030, USA; Department of Biostatistics and Data Science, University of Texas School of Public Health, Houston, TX, USA; Department of Biostatistics and Data Science, University of Texas School of Public Health, Houston, TX, USA; Weatherhead PET Center for Preventing and Reversing Atherosclerosis, Division of Cardiology, Department of Medicine, University of Texas McGovern Medical School, 6431 Fannin St., Room MSB 4.256, and Memorial Hermann Hospital, Houston, TX 77030, USA; Weatherhead PET Center for Preventing and Reversing Atherosclerosis, Division of Cardiology, Department of Medicine, University of Texas McGovern Medical School, 6431 Fannin St., Room MSB 4.256, and Memorial Hermann Hospital, Houston, TX 77030, USA; Weatherhead PET Center for Preventing and Reversing Atherosclerosis, Division of Cardiology, Department of Medicine, University of Texas McGovern Medical School, 6431 Fannin St., Room MSB 4.256, and Memorial Hermann Hospital, Houston, TX 77030, USA

**Keywords:** Chronic stable coronary syndromes, Positron emission tomography, Coronary flow reserve, Coronary flow capacity, Lifestyle modification, Optimal medical therapy, Coronary revascularization

## Abstract

**Background and Aims:**

The randomized CENTURY trial tested the hypothesis that a comprehensive strategy integrating intense lifestyle modification and aggressive medical management to goals with revascularization reserved for severely reduced coronary flow capacity (CFC) by positron emission tomography (PET) would reduce risk factors, subsequent revascularization, death and myocardial infarction (MI) compared with standard of care in chronic stable coronary artery disease (CAD).

**Methods:**

Participants were randomly assigned to standard or comprehensive care groups. Rest-stress PET quantified CFC for physiological CAD severity at baseline, 2, 5, and up to 11 years. The comprehensive care group reviewed PET results with frequent clinic visits and open 24/7 phone/email support. Standard care lacked supportive contact with blinded PET results that were unblinded only for severely reduced CFC with high mortality risk for potential revascularization.

**Results:**

Between 2009–2017, 515 patients were assigned to comprehensive care and 513 to standard care and followed for 5 or more years. Comprehensive vs standard care decreased risk factors and summed 5-year risk score (Δ−1.1 vs + 0.33; 95% confidence interval −1.84 to −0.97; *P* < .0001), decreased cumulative 11-year all-cause death (4.7% vs 8.2%; *P* = .023), death or MI (7.0% vs 11.1%; *P* = .024) late revascularization (9.5% vs 14.8%; *P* = .021) and major adverse cardiac events (20.5% vs 29.9%; *P* = .0006). Only 56 of 1028 (5.4%) CENTURY patients with chronic CAD had revascularization within 90 days predominantly guided by CFC severity.

**Conclusions:**

The randomized CENTURY trial demonstrates that comprehensive integrated lifestyle modification and medical management towards goals with revascularization reserved for severely reduced CFC, significantly reduced risk factor scores, death, death or MI, and revascularization.

**ClinicalTrials.gov:**

NCT00756379

## Structured Graphical Abstract

**Structured Graphical Abstract:**
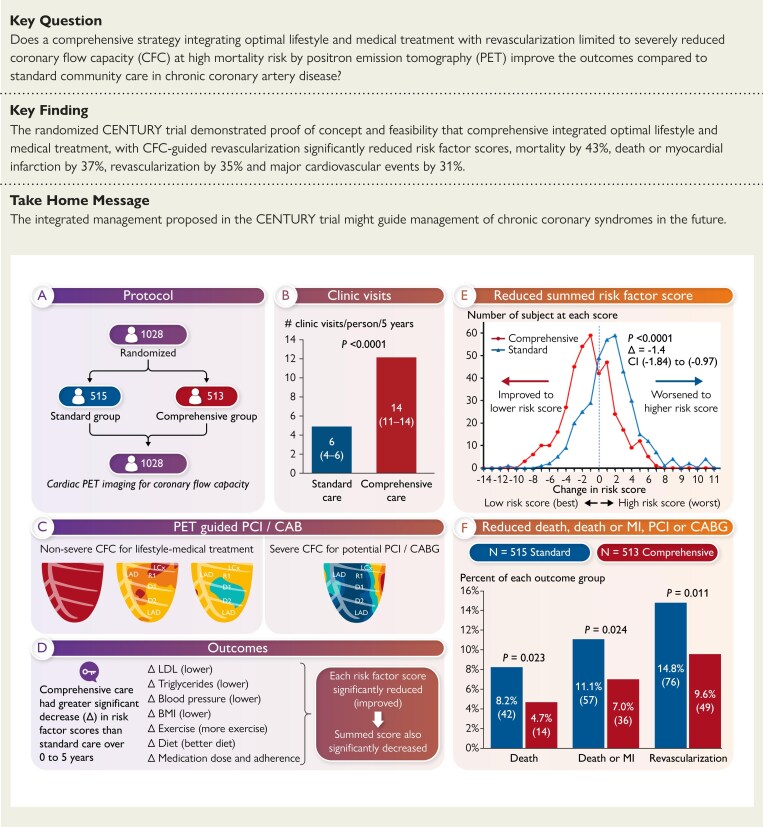

**See the editorial comment for this article ‘A CENTURY of progress: using physiology-based imaging to improve patient outcomes in chronic coronary artery disease’, by M.F. Di Carli and R. Blankstein, https://doi.org/10.1093/eurheartj/ehaf508.**

## Introduction

Landmark randomized trials demonstrated that early coronary revascularization relieved angina but did not reduce mortality or major adverse cardiac events (MACE) over medical therapy, with remaining high annual MACE of 3–4%.^1–5^ In the COURAGE trial, only 3% of patients reached risk factor targets.^1,2^ Suboptimal medical therapy and lifestyle modification were also reported for BARI 2D and FREEDOM trials.^1^ The ISCHEMIA trial showed no mortality benefit.^4^

The FAME trial^5^ used fractional flow reserve (FFR) based on experimental fluid-dynamic equations^6^ of the senior author, to demonstrate safely deferring percutaneous coronary intervention (PCI) in patients with reduced FFR (>0.75–0.8), but no significant reduction in death. No randomized trial is reported for a comprehensive integrated strategy of combined lifestyle, medical treatment and coronary revascularization for severely reduced quantitative myocardial perfusion by positron emission tomography (PET) incurring high mortality risk most likely to have survival benefit by coronary revascularization thereby also reducing non-beneficial procedures.

The CENTURY trial (*C*omprehensive Lif*E*style Modificatio*N*, Optimal Pharmacological *T*reatment and *U*tilizing PET Imaging for Quantifying and Managing Stable Corona*R*y Arter*Y* Disease) (ClinicalTrials.gov NCT00756379) addressed both issues of objective physiologic severity of coronary artery disease (CAD) for guiding revascularization and simultaneous comprehensive integrated lifestyle-medical treatment towards risk factor goals. Since both components are not addressed in prior single treatment design, we randomized patients with chronic stable CAD referred by physicians from their clinical practice to standard care and comprehensive care groups. The latter included frequent, close follow-up support for intense lifestyle modification and aggressive medical therapy towards pre-specified risk factor goals.

After randomization, all participants had stress-rest PET to defer or guide invasive interventions in both groups based on the pre-established threshold of objective, severely reduced coronary flow capacity (CFC) by quantitative PET perfusion imaging associated with high mortality risk that is significantly reduced 54% by revascularization in large non-randomized cohorts.^7–16^ Correspondingly, PETs with non-severe CFC assured patients and physicians on safely deferring or precluding invasive coronary angiograms or procedures in favour of lifestyle-medical management.

Prior randomized trials employed imaging methodologies, invasive techniques, optimal medical treatment, or lifestyle alterations as individual strategies affecting outcomes. In contrast, viewed as a physician interacting with a patient for suspected or known CAD, the randomized CENTURY trial tested two related hypotheses. First, the comprehensive group undergoing integrated lifestyle-medical treatment would have greater reduction in a comprehensive summed score comprised of risk factors, better laboratory test results and compliance with medications, dietary modification, weight control, and exercise. Second, as a corollary hypothesis, the comprehensive group with lower comprehensive summed risk score would have better outcomes including less death, non-fatal myocardial infarction (MI), late revascularization and their combination as MACE than the standard group.

## Methods

### Study design

**Figure 1:**
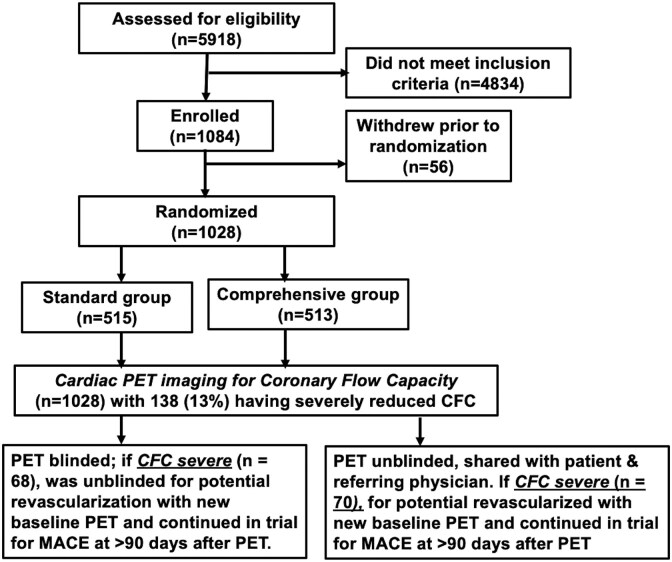
Trial profile. The participants are the population presenting to the 651 physicians referring patients to the trial due to high-risk factors, suspected or known coronary artery disease, with or without symptoms

**Figure 2:**
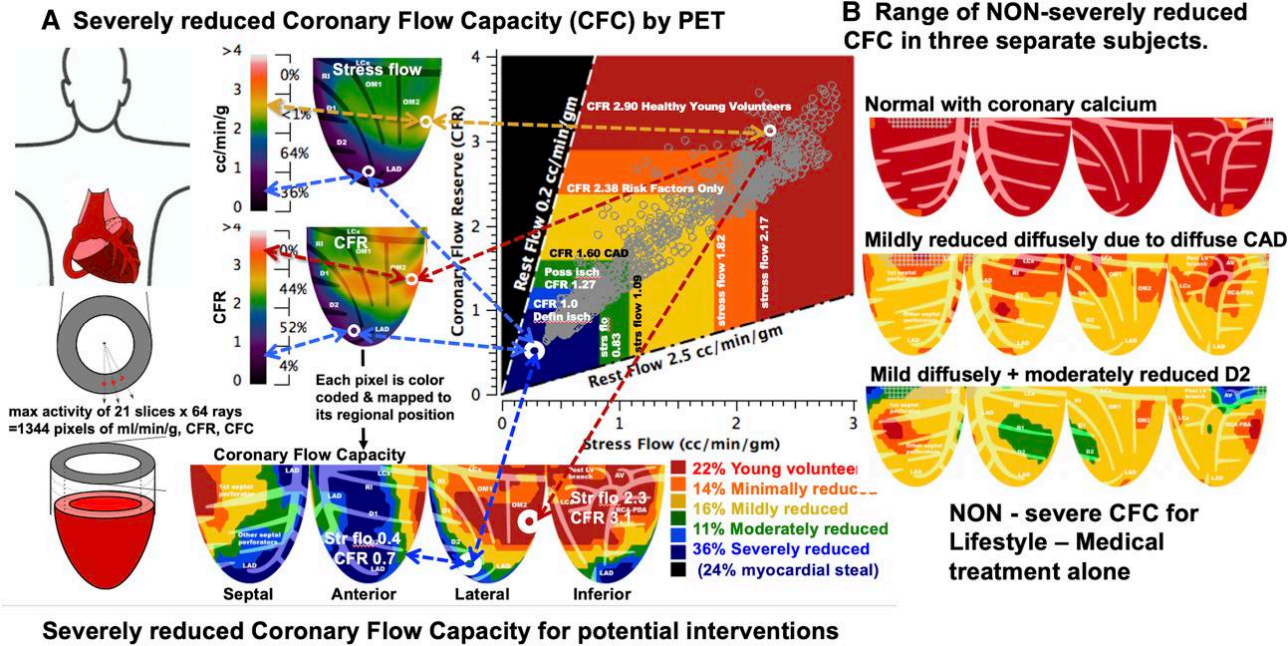
Positron emission tomography quantitative analysis. (*A*) Schematic of rest and stress mL/min/g, their ratio as coronary flow reserve and the combination of stress mL/min/g and coronary flow reserve as coronary flow capacity per regional pixel by positron emission tomography mapped for the left ventricle as percent of left ventricle in each severity range (see text and Supplementary data online, *Figure S1* for additional details). Example of severely reduced coronary flow capacity map (blue) incurring high mortality risk for which coronary angiogram and potential revascularization may be appropriate depending on clinical circumstances, clinical judgement and informed patient consent. The automated processing for a pixel with severely reduced coronary flow capacity is shown having stress flow (perfusion) of 0.4 mL/min/g plus coronary flow reserve 0.7 (myocardial steal); 36% of left ventricle consists of pixels having stress mL/min/g ≤ 0.8 plus coronary flow reserve ≤1.2 colour-coded blue. For comparison, another pixel has normal stress perfusion of 2.3 mL/min/g plus coronary flow reserve of 3.1 and 22% of left ventricle having pixels with stress mL/min/g ≥ 2.17 and coronary flow reserve ≥2.9. All colour-coded thresholds were objectively determined by ROI analysis of prior large cohorts and healthy young volunteers in clinically pre-defined colour-coded groups shown in the colour-coded map of *Figure 2A*. (*B*) Non-severe coronary flow capacity maps are associated with relatively low risk for which lifestyle-medical treatment is indicated without invasive interventions. PET, positron emission tomography; CFR, coronary flow reserve; CFC, coronary flow capacity; LV, left ventricle

CENTURY is a randomized, blinded, controlled, single-centre trial from the University of Texas McGovern Medical School and Memorial Hermann Hospital, Houston, Texas. This large single-centre study was approved by the UT Committee for the Protection of Humans as summarized in *Figure 1*. Trial design and methodology as previously reported^14^ were based on information from other large non-randomized studies.^7–16^ After signing informed consent and undergoing quantitative PET for quantitative myocardial perfusion (*Figure 2*), participants were randomized to either standard community care by their referring physicians or to comprehensive aggressive lifestyle and medical management towards pre-specified risk factor targets motivated by PET scan results, frequent clinic visits and contact with the CENTURY team in collaboration with referring physicians.

Quantitative PET perfusion in this randomized trial was not aimed at routine screening for CAD but for deferring or precluding coronary revascularization procedures paralleling invasive FFR but using non-invasive, comprehensive, physiologic measures of severity.^6^ Based on prior non-randomized cohorts,^7–18^ severely reduced CFC (*Figure 2*) non-invasively selected the minimum proportion of participants at a pre-defined, severe threshold (pixels with CFR ≤1.27 and stress perfusion ≤0.83 mL/min/g) (see Supplementary data online, *Figure S1*) at high risk of mortality warranting revascularization depending on clinical judgement and patient preference.

### Participants, study randomization and masking

Patients aged ≥40 years with high-risk factors, subclinical, suspected or established CAD were recruited from physician and patient referrals for signed consent, characterized in *Table 1*. Inclusion and exclusion criteria are listed in Supplementary data online, Table  *S1* as previously reported.^14^ A computer-based algorithm was used for randomly assigning participants to standard care or comprehensive care groups. There were no significant differences between the two groups at baseline indicating successful randomization (*Table 1*).

**Table 1:** Baseline characteristics of participants

Characteristics	Comprehensive care (*n* = 513)		Standard care (*n* = 515)	
Age (years)	61 ± 9	61 (55–67)	61 ± 9	61 (55–67)
BMI (kg/m^2^)	29 ± 3.6	29 (26–32)	28.9 ± 3.8	29 (26–32)
Male sex	344 (67)		353 (69)	
Hx_hypertension	441 (86)		426 (83)	
Hx_dyslipidaemia	507 (99)		509 (99)	
Hx_diabetes mellitus	353 (69)		326 (63)	
Hx_smoking	378 (74)		398 (77)	
Hx_CAD (family)	266 (52)		293 (57)	
Hx_MI, revascularization + angiography	190 (37)		205 (40)	
Risk factors	322 (63)		310 (61)	
Statin	354 (69)		381 (74)	
Antiplatelet	354 (69)		352 (68)	
Beta-blocker	212 (41)		219 (43)	
ACEI or ARB	322 (63)		304 (59)	
Cal Channel Blocker	98 (19)		95 (18)	
Diuretic	142 (28)		144 (28)	
CAC >120 HU	396 (77)		419 (82)	
Mild CFC >15% LV	219 (43)		205 (40)	
Severely reduced CFC	69 (14)		70 (14)	
Stress EF	71 ± 8.7	72 (67–77)	71 ± 9.2	72 (67–77)
CFR Min quadrant avg	2.4 ± 0.6	2.3 (1.9–2.7)	2.4 ± 0.6	2.4 (2.0–2.8)
Stress mL/min/g MQA	1.8 ± 0.6	1.8 (1.4–2.2)	1.9 ± 0.7	1.8 (1.4–2.3)
Visual abnor relative stress	175 (34)		158 (31)	
CFR global average	2.6 ± 0.6	2.5 (2.2–3)	2.6 ± 0.6	2.6 (2.2–3.0)
Stress mL/min/g global	2.0 ± 0.6	2.0 (1.6–2.4)	2.1 ± 0.7	2.0 (1.6–2.5)
Stress mL/min/g max	2.7 ± 0.7	2.6 (2.2–3.2)	2.8 ± 0.8	2.7 (2.2–3.2)
CFR max in LV	3.6 ± 0.8	3.5 (3–4.1)	3.6 ± 0.9	3.5 (3.0–4.1)
PET stress angina^a^	32 (6)		53 (10)	
PET stress STΔ^a^	79 (15)		75 (15)	
Exer treadmill METS	10.6 ± 2.8	10.2 (9–14)	10.6 ± 2.8	10.2 (9–14)
ETT STΔ > 1 mm	97 (19)		103 (20)	
ETT angina *P* = .02	15 (3)		30 (6)	

^a^During PET dipyridamole stress.

Values are given as *n* (%), mean ± standard deviation, or median (upper and lower quartile).

ACEI, angiotensin-converting enzyme inhibitor; ARB, angiotensin-receptor blocker; CAC, coronary artery calcium; CAD, coronary artery disease; CCB, calcium channel blocker; CFC, coronary flow capacity; CFR, coronary flow reserve; Ch^2^, Chi-squared test; EF, ejection fraction; HU, Hounsfield units; Hx, history of; LV, left ventricle; MQA, minimum quadrant average; PET, positron emission tomography; Visual abnormal relative stress, visual abnormal regional relative stress perfusion defect (≤60% of max for ≥10% of LV); STΔ, ECG ST depression during dipyridamole stress PET imaging; ETT, exercise treadmill test by Bruce protocol; METs metabolic equivalent of task.

No significant differences by Chi-square test. *t*-test or Wilcoxon test except for lowest row for slightly more angina during ETT in the standard care group compared with comprehensive group.

Of 651 physicians referring patients to CENTURY, each of top 10 referring physicians enrolled 1.0%–3.7% of 1028 randomized patients, and remaining 641 physicians referred <1% each. Large numbers of potential participants were screened for each successful recruitment (*Figure 1*) for several reasons. Participation requested a minimum 5-year commitment, random assignment to lifestyle-medical modification, serial follow-up supporting management and PET imaging by the CENTURY team that incurred some patient and physician resistance in the highly competitive Houston heart care community by cardiologists having office SPECT scanners.

### Lifestyle modification and medical management

For the comprehensive care group, frequent clinic visits motivated adherence to personalized dietary modification, weight control, exercise regimes, compliance with maximal medication dosing, and reassurance of non-high-risk CAD suitable for medical management without invasive interventions by a pre-established PET-CFC threshold based on prior nonrandomized reports.^7–16^ The team specifically trained for the CENTURY trial included six cardiologists, three nurses, three nutritionists, four research assistants, three technologists, five communications, and management staff contacting participants in the comprehensive care group at 1- to 6-month clinic visits, and by 24/7 phone accessibility, email or letters as individually needed for optimal adherence to healthy living and medications.^14^

As summarized in *Table 2*, for every comprehensive care follow-up clinic visit, the nutritionist, nurse and cardiologist counselled each participant over 1.5–2 h to review all interim data for every risk factor and steps or treatment for achieving specific goals of diet, exercise, weight, % body fat, medication adherence, optimal medications and dose towards blood pressure, lipid, diabetes goals, and treatment of comorbidities. The specific goals for each of 10 modifiable risk factors are detailed in Supplementary data online, *Tables S2*  to  *S4* as previously reported in the methods paper^14^ reviewed briefly here. The baseline, 2- and 5-year visits lasted for 4 h, with review of treadmill test and PET scan results in addition to the nurse, nutritionist, and cardiologist sequence. For the comprehensive group, a dedicated CENTURY phone was extensively used for immediate call-in questions or support with 24/7 message answering for next-day-call-back by a nutritionist, nurse, or cardiologist. Frequency of contact was systematically recorded with time, topic, and response by CENTURY staff. Written reports with colour-coded risk factor trends were sent to each participant and referring physician after every clinic visit. Participants were encouraged to report their success or difficulty in achieving risk factor goals as ongoing support towards developing healthy living habits.

**Table 2:** Components of comprehensive group management

Complete medical history, procedures, risk factors, medications, and exam. Lab tests: lipid profile, chemistry profile, CBC, HbA1c, BMI, and % body fat. Dietary history by certified nutritionist completing the Food Frequency Questionaire and 3-day food Diary using Nutritionist Pro software. Exercise history type, duration, frequency, and intensity. Bruce treadmill test. Rest-stress PET scan images for regional perfusion in mL/min/g, coronary flow reserve, coronary flow capacity as % of left ventricle. *Review all results with each participant, every risk factor, risk of adverse events and specific steps for improving each risk factor and refering MD.* Review visual colour-coded PET scans with for individual and referring MD. Consulting with CENTURY Cardiologist, Nurse and Nutritionist for individualized Diet, Weight, Exercise goals, medications. Optimize all medications and doses towards specific BP, lipid, diabetic goals, or symptoms. All of above done over 3 to 4-h visit by nurse, cardiologist, nutritionist. Follow-up CENTURY clinic every 2–6 months depending on adherence. At every follow-up clinic, repeat all labs & consult steps above but not ETT/PET. Participants have online access and dedicated phone access to cardiologist, nurse, or nutritionist 24/7 for advice or medical assistance. At every clinic visit, participants review every risk factor, improvement steps, and receive a written colour-coded schematic summarizing successes, remaining goals for improvement and instructions for reporting back corrective steps over the following month. Follow-up ETT and PET at 2 and 4 years with personal review of results as motivation for continued or improved adherence of every risk factor. *Standard group management* The same as the comprehensive group down to but no review of any results and no follow-up with CENTURY staff. ⇩ *No review of results* Participant returned to referring physician for standard care in community practice. ⇩ At 2 and 5 years, the same complete medical history, exam, labs, BMI, % body fat, dietary and exercise assessment, treadmill test and rest-stress quantitative PET perfusion. No review of results or PET images with participant or referring MD. No medical contact with CENTURY staff. No follow-up clinic visits. No 24/7 dedicated access to CENTURY staff.

In the comprehensive group, PET images were reviewed with participants and their physicians. In the standard care group, patients and referring physicians were blinded to PET results. However, severely reduced CFC incurs high mortality risk that is decreased significantly after revascularization compared with medical management alone for similar CFC severity in prior reports^7–16^ (*Figure 2A*) (more detail in Supplementary data online, *Figure S1*). Consequently, for patients with severely reduced CFC (pixels with CFR ≤1.27 and stress perfusion ≤0.83 mL/min/g expressed as % of the left ventricle [LV]) (*Figure 2A*) in comprehensive and standard care group at baseline or during the 5-year study, the severe PET results were reviewed with participants and their physician (breaking the blind for the standard care group) with a written report recommending coronary angiography and potential revascularization depending on clinical judgement and individual clinical circumstances.

American Heart Association healthy living guidelines were reviewed at the baseline visit and returned to their referring physician for standard care without follow-up appointments or support contact by the CENTURY team (*Figure 1*). At baseline, 2- and 5-year clinic visits, risk factor assessment, PET scans and treadmill testing were acquired with review of results or supporting consultation in the comprehensive but not the standard groups.

### Protocol visits, quantitative risk factor scoring, patient guidance and treatment towards each risk factor goal

Although primary or secondary preventative measures are well known, prior non-randomized studies suggested that a more comprehensive, integrated, long-term supportive approach was necessary for influencing hard outcomes.^7–16^ Therefore, scores for 16 individual risk factors each in 5 grades of objective severity and a cumulative single summed risk score were determined for each participant at every visit (see Supplementary data online, *Tables S2*–*S4*).^14^ Risk factor scores were assessed by blinded staff at baseline, during each follow-up visit for comprehensive care group, and at baseline and 5 years for the standard care group including the following: Low-density lipoprotein, high-density lipoprotein, triglycerides, glycated haemoglobin (HbA_1c_), history of diabetes mellitus (additional to HbA_1c_), blood pressure, smoking status, body mass index (BMI), % body fat, metabolic equivalents (METs) on treadmill by Bruce protocol, component and summed diet scores, adherence to medications, age, gender, family history of premature CAD, coronary artery calcium (CAC) score (performed during PET-CT imaging), or known CAD (see Supplementary data online, *Table S2*).^14^

At patient visits, management decisions were made by one team member of CENTURY staff in comprehensive care group who was software-blocked from entering endpoint data into the database that was done by other blinded members of the team for each visit who had not advised the participant.^14^ Separately from the registered nutritionist advising participants at each clinic visit, another blinded nutritionist entered dietary data (see Supplementary data online, *Table S3*) at baseline, at every protocol clinic visit, and at the final 5-year clinic visit (see Supplementary data online, *Table S4*).^14^ For completeness, dietary histories were obtained by two separate tools, the Food Frequency Questionnaire entered in the National Cancer Institute website and the 3-day Food Diary entered using *Nutritionist Pro* and exported using the dedicated Data Extraction Tool.

Exercise treadmill testing (ETT) was carried out using the Bruce protocol at baseline, 2 and 5 years with quantitative assessment of exercise capacity represented as METs,^14^ and an exercise component risk score was calculated. Achieving ≥10 METs on ETT incurred a zero exercise risk score increasing to the worst score of 3 for achieving <5 METs (see Supplementary data online, *Table S2*).

### Quantitative positron emission tomography myocardial perfusion imaging, coronary flow capacity and physiological severity of coronary artery disease

After randomized assignment to comprehensive or standard care groups, quantitative myocardial perfusion was measured in all participants (*Figure 2*) per protocol. CFC by PET established the less-than-severe CFC threshold for safely deferring or precluding invasive procedures in favour of lifestyle-medical management (*Figure 2B*), or severely reduced CFC indicating potential angiogram or revascularization (pixels with CFR ≤1.27 and stress perfusion ≤0.83 mL/min/g expressed as % of LV) (*Figure 2A*). Quantitative PET determined rest-stress mL/min/g, CFR and CFC (*Figure 2* and Supplementary data online, *Figure S1*) at baseline, and during follow-up at 2 and 5 years. Additional PET scans were performed between these protocol schedules if clinically indicated.

Absolute rest and stress myocardial perfusion in mL/min/g were quantified for each of 1344 pixels of LV images derived from maximal activity of Rb-82 along 64 radii for each of 21 short-axis slices.^7–16^ This maximal regional myocardial activity optimized quantitative myocardial perfusion per regional pixel of specific coronary arteries down to tertiary branches. It thereby avoids averaged perfusion in assumed arterial distributions or externally imposed arbitrary regions of interest overlapping multiple adjacent arterial distributions that may not reflect actual artery-specific distribution, severity, or size at risk (*Figure 2*). Myocardial perfusion in mL/min/g per pixel distribution has been validated experimentally and clinically for the HeartSee software (FDA K202679)^7–16^ for long-term outcomes with and without revascularization in large non-randomized studies (*Figure 2*) as the basis for CENTURY design.^7–16^ Our coefficient of variance for perfusion in mL/min/g for humans is ±10% on serial rest-rest and on stress-stress images in the same patient acquired minutes apart under stable physiologic conditions.^16^ Day-to-different day variability is ±20% due to minute-to-minute methodologic variability of ±10% plus day-to-different-day biological variability of ±10%.^16^ Rest and stress perfusion in mL/min/g and CFR are combined into CFC per colour-coded pixel map arterial-specific distribution expressed as %LV for each colour-coded range of CFC.

*Figure 2A* illustrates two different pixels—one pixel with severely reduced CFC colour-coded blue and one pixel with normal CFC colour-coded red. All other pixels are correspondingly colour-coded for minimal (orange), mild (yellow) and moderate (green) reductions in CFC based on the receiver operating characteristic analysis of threshold severities in large cohorts as previously reported.^7–14,16^ Each colour-coded pixel over each range of severity is mapped as % of all pixels comprising the LV shown by the colour-coded CFC map and its colour bar scale, thereby providing artery-specific size-severity of CFC. The colour histogram bar scales represent pre-defined ranges of normal CFC (*red*), minimally reduced CFC (*orange*), mildly (*yellow*), moderately (*green*), and severely (*blue*) reduced CFC.^7–14,16^ CFC maps with any pixels having both CFR ≤1.27 and stress perfusion ≤0.83 mL/min/g are defined as severely reduced CFC and colour-coded *blue* based objectively on ROC analysis for highest likelihood of definite angina and significant STΔ during dipyridamole stress requiring aminophylline reversal by region of interest analysis.^7–16^ CFC histogram distribution maps on serial paired PET scans in the same patient on the same or different days show no differences on the Kolmogorov–Smirnov test for differences in histogram distributions.^16^

For the comprehensive group, every PET consultative report included quantitative images of artery-specific, size-severity of relative perfusion defects and perfusion metrics including regional rest, stress mL/min/g, CFR and CFC maps. Most importantly, for all PET consultative reports, quantitative perfusion metrics were integrated and interpreted within the perspective of detailed medical history of clinical symptoms, particularly typical or atypical angina, risk factors, age, comorbidities, history of MI, coronary procedures, status of medical treatment by all medications, remaining risk factors, mobility, comorbidities and patient preferences. All PET images and their clinical interpretation were reviewed with each participant in the comprehensive care group and with the referring physician followed by a written report with colour-coded PET images to both. For the standard care group, PET results were not reported to referring physicians or reviewed with participants unless showing severely reduced CFC for which the blind was broken, and report was sent to referring physicians and reviewed with participants with suggested angiogram and potential revascularization.

### Study outcomes

The primary outcome is change in summed risk score from baseline to 5 years, and secondary outcomes of MACE and its separate components of all-cause death, death or non-fatal MI, stroke, and late revascularization at >90 days after baseline PET scan. Revascularization at ≤90 days after baseline PET or during routine protocol scheduled follow-up PET was counted as elective guided by baseline PET as opposed to a late event of acute revascularization or severe progression by PET during study follow-up.

### Statistical analysis, electronic database and trial design

Outcome data were analysed by intention-to-treat, using *t*-test of differences between comprehensive care and standard care groups for continuous variables and *χ*² test for binary variables. SAS 9.4 and R 4.4.1 were used for analyses of times to death and MACE by Kaplan–Meier plots and Cox regression modelling and by the win ratio analysis with priority order of death > MI > stroke > revascularization accounting for the event times.^17^ The null hypothesis was no difference between standard care and comprehensive care groups. Comprehensive statistical analysis were carried out including *χ*² for relative risk reduction, Kaplan–Meier plots, Cox regression modelling and the win ratio to assure statistical validation of outcomes of CENTURY’s comprehensive integrated strategy of quantitative imaging-lifestyle-medical Rx-interventions not previously reported. The intent was to avoid divergent criticism of relying on any single statistical analysis. For additional blinding, CENTURY staff involved in patient management were software-blocked from entering objectively measured or reported data. Blinded, independent nurses from University of Texas Center for Clinical and Translational Sciences entered objective data into CENTURY database. All PET data were automatically quantified by blinded technologists and analysed by blinded statisticians.

An electronic medical record and searchable, relational database, compliant with Health Insurance Portability and Accountability Act, was developed over one year using the FileMaker Pro platform (Claris International, Santa Clara, CA, USA).^14^ All data entries were software security restricted to specific blinded personnel separate from blinded enterers for medical management. Sample size of 1028 associated with 86% power for primary and secondary endpoints accounting for declined consent and loss to follow-up as reported in the Methods paper.^14^ Based on extensive prior non-randomized studies,^7–16^ a minimum 5-year long randomized trial design was necessary for determining mortality with comprehensive lifestyle-medical management and interventions reserved for severe quantitative PET perfusion abnormalities.

## Results

### Characteristics of trial participants

From a total of 5918 patients assessed for eligibility, 513 patients were randomly assigned to the comprehensive care group and 515 to standard care group between 5 March 2009, and 13 April 2017 (*Figure 1*). For 1028 patients enrolled at baseline, prevalence was high for known or suspected CAD, and risk factors in a population seen in cardiology practice of referring physicians. All had some baseline PET-CT abnormality including 89% with coronary calcium, 72% with reduced stress relative subendocardial perfusion,^7^ 14% with severely reduced CFC (*Figure 2A*) as compared with non-severe CFC (*Figure 2B*), 38% with known clinical CAD as evidenced by cardiac events or angiography, and 99% with dyslipidaemia and other risk factors (*Table 1*). At baseline, all participant characteristics and PET perfusion metrics were similar between the comprehensive and standard care groups (*Table 1*). Final 5-year follow-up was completed on 28 April 2022, with additional follow-up continuing up to over 11 years after randomization for patients recruited early or wishing to continue after they signed consent for extended follow-up.

### Change in summed risk factor scores on follow-up

**Figure 3:**
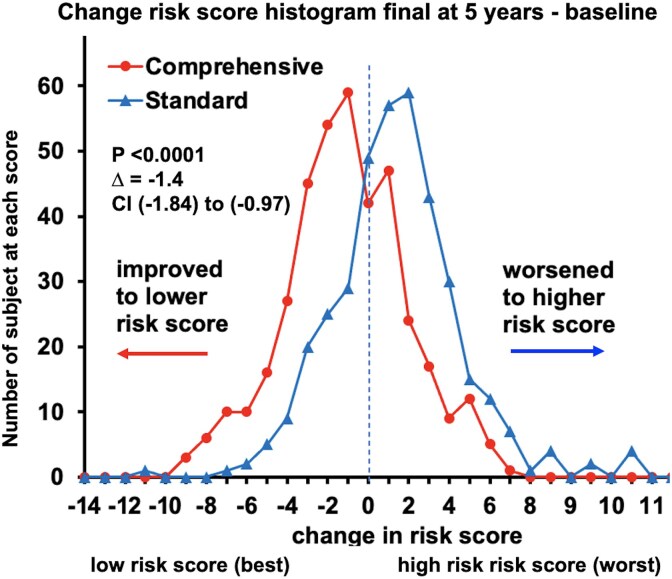
Outcomes—risk factor change. The cumulative or summed risk score change (Δbaseline-5year summed risk score) for comprehensive care vs standard care groups. The (−) sign indicates a smaller or lower risk score vs a (+) risk score as being higher risk. At baseline, the summed risk score of comprehensive and standard groups are similar (see Supplementary data online, *Figure S2*). At 5-year follow-up, risk scores for the comprehensive care group move left towards lower risk, whereas scores for the standard care group move right towards higher risk (see Supplementary data online, *F*igure  *S2*)

At 5-year follow-up, the summed risk score histogram shifted leftward with improved (lower) risk scores for comprehensive care group and rightward or worse (higher) summed risk score for the standard care group for a significant delta (Δ) between the groups (*Figure 3*, Supplementary data online, *Tables S5*, *S6* and *Figure S2*). There was significant inter-group comparative difference in baseline to 5-year delta (Δ5year-baseline) changes. The baseline to 5-year change in component risk score and for each risk factor individually was significantly better (lower) for comprehensive care compared with standard care group for LDL, triglycerides, blood pressure, BMI, exercise, diet, and medication adherence (*Table 2*). The summed risk score improved in 70.3% of patients compared with 52.8% in standard care group (see Supplementary data online, *Table S6*). Sensitivity analysis accounting for incomplete risk scores due to patients missing follow-up clinic appointments did not change these conclusions (see Supplementary data online, *Table S5*). This risk score improvement supports the first hypothesis that the comprehensive care group would achieve significantly better risk factor control than the standard group.

### Mortality or major adverse cardiac events and all-cause death

**Figure 4:**
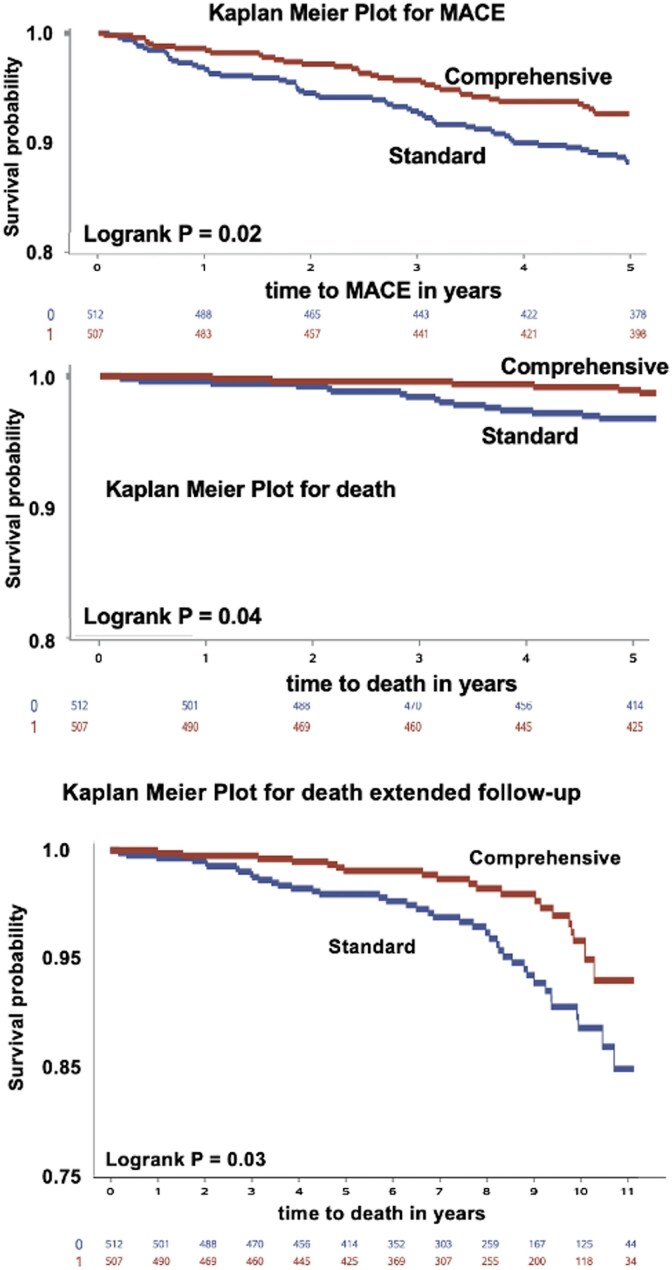
Kaplan–Meier survival plots for time to major adverse cardiac events or death. All 5-year analyses show significantly improved survival and reduced major adverse cardiac events in the comprehensive care group compared with the standard care group. Major adverse cardiac events. Kaplan–Meier and Cox regression modelling for death at up to over 11 years after randomization shows progressive divergence of survival probability favouring improved survival in the comprehensive over the standard care group. Cox regression modelling for time to major adverse cardiac events of death is in Supplementary data online, *Figure S3*. MACE, major adverse cardiac events

Kaplan–Meier (*Figure 4*) and Cox regression modelling (see Supplementary data online, *Figure S3*) with survival plots for time to death or time to MACE over 0–5 years showed significantly fewer events in the comprehensive care vs standard care group. Due to co-linearities among all risk factors, univariate Cox regression modelling used assignment to comprehensive or standard cared group as the independent variable. At 5 years, comprehensive vs standard care MACE was 35/515 (6.8%) vs 58/513 (11.3%) (*P* = .01) and deaths were 6/515 (1.17%) vs 16/513 (3.1%) (*P* = .03). Events after 5 years were censored.

For extended follow-up (*Figure 4*), the comprehensive vs standard group had progressively divergent probability of survival favouring comprehensive care over standard care up to over 11 years after randomization. Various multivariable Cox regression analysis were attempted but multiple co-linearities among all the individual risk factors required univariable Cox regression modelling using group assignment to comprehensive or standard care as the independent variable (see Supplementary data online, *Figure S3*).

**Figure 5:**
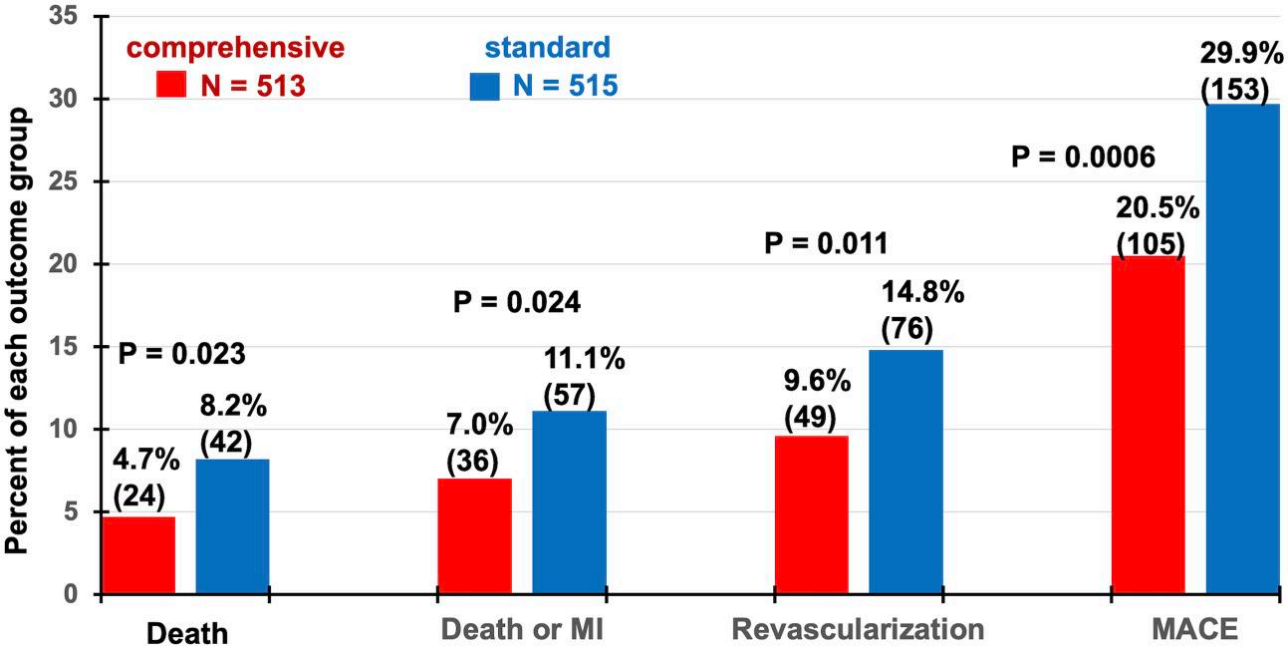
Cumulative all-cause mortality, death or non-fatal myocardial infarction, revascularization and major adverse cardiac events. Over extended follow-up these outcomes are significantly lower in the comprehensive care than in the standard care group (χ^2^ analysis) paralleling Kaplan–Meier and Cox regression modelling over extended follow-up in the prior figure

*Figure 5* shows cumulative events up to over 11 years of all-cause death, death or MI, revascularization at >90 days after PET and MACE over extended follow-up. Prevalence of all outcome events was significantly lower in comprehensive than standard care group. Corresponding relative reductions in comprehensive vs standard care groups were 42.7% for death, 37.0% for death or MI, 35.1% for revascularization and 31.4% for MACE. Excluding all revascularizations from analysis also did not change these differences in hard outcomes or their statistical significance between comprehensive care vs standard care groups. The win ratio^17^ for MACE with priority order of death > MI > stroke > revascularization accounting for event times demonstrated significantly fewer MACE in comprehensive care compared with standard care group (win ratio 1.51, 95% CI 1.09–2.09, *P* = .01). These outcomes confirm the hypothesis that the comprehensive care group would have fewer adverse events than the standard care group.

**Figure 6:**
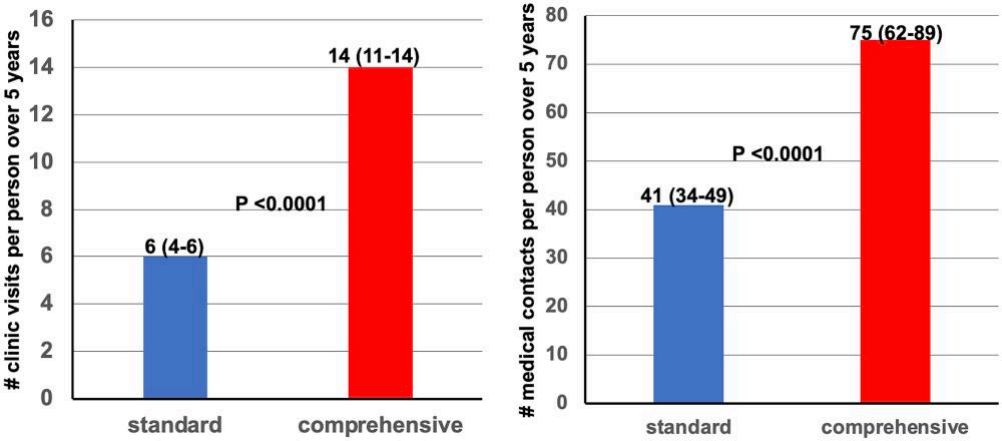
Frequency of medical contacts between participants and CENTURY staff by clinic visit, phone, email or letters related to lifestyle, medications or symptoms with 24/7 access. *N* = 57 877 for 1028 participants over 5 years. As per protocol, supportive clinic visits by the comprehensive group were significantly more frequent than for the standard group. The bar graph shows medians (upper and lower quartiles) with Wilcoxon *P*-values for the number of clinic visits and the number of medical contacts for standard vs comprehensive groups

As per protocol, frequency of clinic visits (*Figure 6*) and frequency of participant contact with CENTURY staff were substantially greater for comprehensive vs standard care group as needed or requested by participants for additional clinic visits, phone access 24/7 by dedicated line, email, or fax for supporting risk factor goals over 5 years.

### Exercise test outcomes

For the 1028 participants, exercise capacity quantified as METs significantly improved in the comprehensive care compared with standard care group (*P* = .002) (*Table 3*). In a subset of 2467 per protocol-paired ETT and PET in same patient on same day or within 2 weeks, neither ETT nor dipyridamole-induced angina or STΔ predicted moderate or severely reduced CFC (green or blue) ≥ 0.5% LV (*χ*² = 3.4, *P* = .07). Diagnostic accuracy of either exercise or dipyridamole-induced angina or ST depression >1 mm for predicting moderate or severely reduced CFC (green or blue) ≥ 0.5% LV were not significantly different at 64% for ETT and 57% for dipyridamole stress.

**Table 3:** Baseline to 5 years change (Δ) in modifiable risk score for each risk factor as defined in Supplementary data online, *Table S2*

Modifiable risk factors	Comprehensive care		Standard care		Between group	
	Mean Δ in risk score	SD of change Δ	Mean Δ in risk score	SD of change Δ	*T*-value of difference in change	*P* for Δ
LDL	−0.459	1.184	−0.008	1.202	−5.234	<.00001
HDL	−0.046	0.567	−0.029	0.566	−0.417	.677
Triglycerides	−0.149	0.783	−0.029	0.812	−2.074	.038
HbA_1c_	0.187	0.750	0.194	0.806	−0.114	.909
BP	−0.026	0.928	0.218	0.950	−3.586	.000
BMI	−0.187	0.473	−0.072	0.464	−3.416	.001
Exercise score^a^	−0.003	0.584	0.127	0.596	−3.049	.002
Diet score^b^	−0.113	0.606	−0.027	0.587	−2.004	.046
Tobacco	−0.067	0.492	−0.050	0.592	−0.416	.678
Medication use	−0.208	0.998	0.003	0.926	−3.025	.003
Sum score	−1.074	3.016	0.328	3.077	−6.36	<.00001

^a^Exercise score is based on METs (metabolic equivalents) of the Bruce protocol treadmill test from Supplementary data online, *Table S2*.

^b^Diet score from Supplementary data online, *Table S3*.

The negative change in modifiable risk score, (−)Δ5 year-baseline, indicates a significant change towards lower or small risk score in the comprehensive compared with the standard group for seven of ten risk score components.

BMI, body mass index; BP, blood pressure; HbA_1C_, glycated haemoglobin; HDL, high-density lipoprotein; LDL, low-density lipoprotein; SD, standard deviation.

### Quantitative perfusion metrics from baseline to 5 years

**Figure 7:**
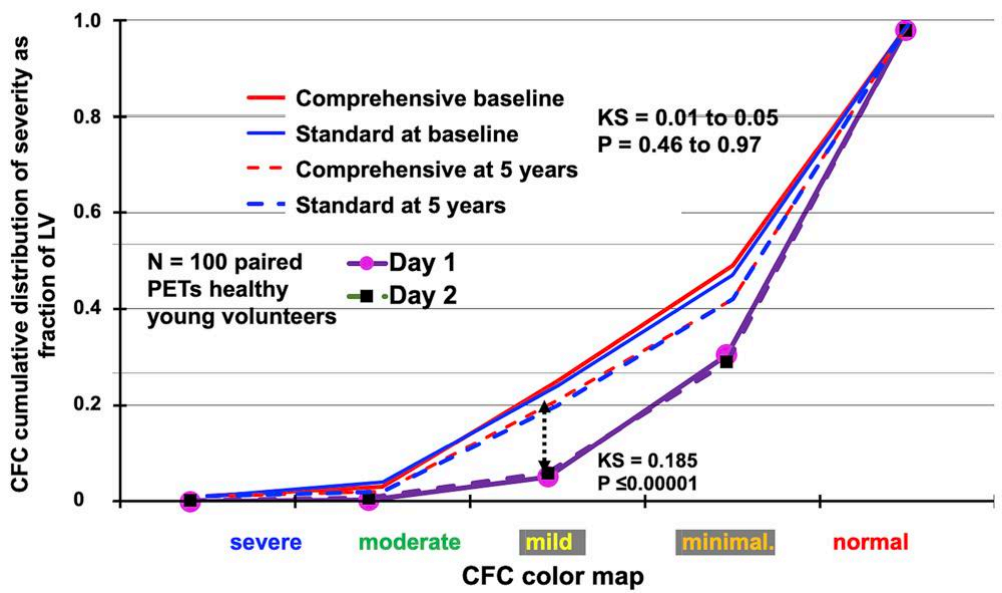
Quantitative perfusion metrics—baseline to 5 years. The Kolmogorov–Smirnov test for coronary flow capacity cumulative histograms showed no differences between comprehensive and standard care groups at baseline, at five years or for baseline to 5-year changes. Similarly, there were no differences between comprehensive vs the standard care group for stress mL/min/g or coronary flow reserve at baseline or 5 years. As a normal reference, the coronary flow capacity Kolmogorov–Smirnov plots for two serial coronary flow capacity maps in the same person for 100 healthy young volunteers without risk factors (not in the trial) shows precise reproducibility that is significant less severe than for participants in this trial. KS, Kolmogorov–Smirnov

No significant differences between comprehensive care and standard care groups were observed for baseline quantitative PET metrics (*Table 1*) or for patients with severely reduced CFC at any time throughout the study (see Supplementary data online, *Table S7*). There were no significant differences between comprehensive care and standard care groups for rest or stress mL/min/g, CFR, or CFC at baseline, at 5 years, or the 0 to 5-year change by Kolmogorov–Smirnov test (*Figure 7*) that are significantly worse than for 100 healthy young volunteers without risk factors (not in the trial).

PET images and quantitative metrics substantially influenced selection of patients for revascularization within 90 days after baseline PET as non-acute, elective, PET-guided intervention depending on integrating all clinical circumstances and clinical judgement of the cardiologist PET reader and referring physician (Supplementary data online, *Table S8*) as detailed in Methods. Fifty-six of 1028 participants (5.4%) had revascularization at ≤90 days driven predominantly by baseline severity of relative PET perfusion defects, severely reduced CFC and quantitative perfusion metrics (*Figure 2A*), history of typical or atypical angina, definite angina or ST depression ≥1 mm during PET stress imaging, age, and male gender that were significantly more common than for participants with no revascularization (see Supplementary data online, *Table S8*). Revascularization after 90 days was categorized as progressive disease or acute coronary syndromes and counted as MACE at >90 days after PET. Of 1028 participants, 19 (1.8%) had an angiogram within 90 days without revascularization—12 in the standard and 7 in the comprehensive group. Therefore, of 75 patients with angiograms, revascularization was performed in 56 (75%) (see Supplementary data online, *Table S8*).

**Figure 8:**
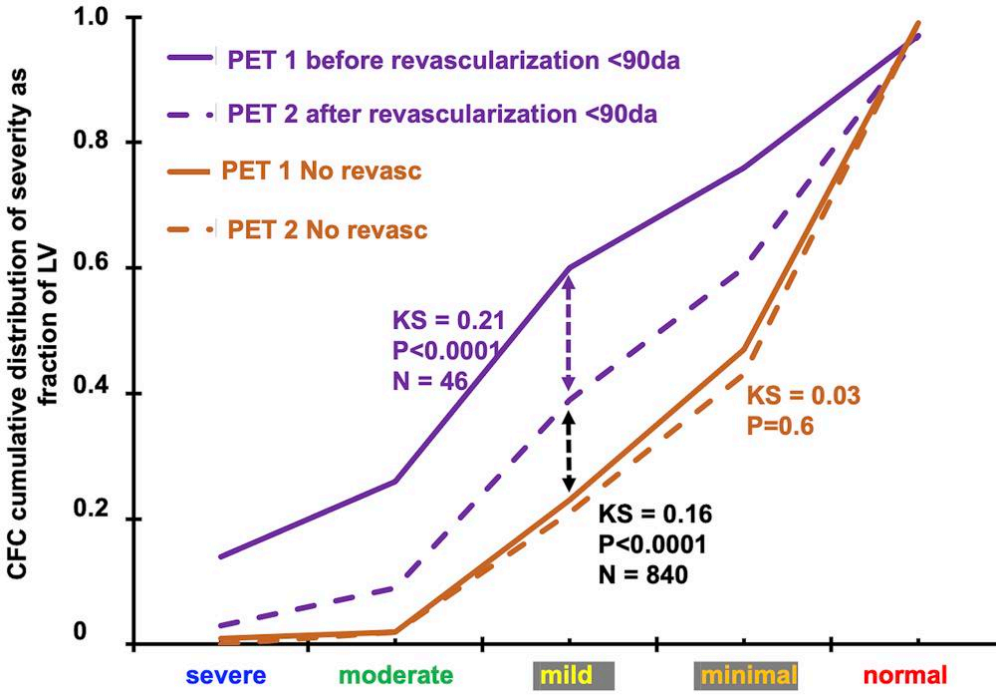
Revascularization. For participants undergoing revascularization at any time during the 5-years, coronary flow capacity before revascularization was more severe than without revascularization, significantly improved after revascularization but remained more severe after revascularization than participants without revascularization

PET before revascularization had predominantly severely reduced CFC that improved after the procedure. However, post-procedure CFC remained significantly worse than CFC of participants without revascularization due to residual CAD (*Figure 8*). For the revascularized group, death and death or MI were significantly greater than in the non-revascularized group (see Supplementary data online, *Table S8*) due to more severely reduced CFC that improved after revascularization but remained worse than the non-revascularized group due to residual diffuse CAD (*Figure 8*). For all participants in both groups, 23.6% (*n* = 243) had severely reduced CFC for which 46/243 (19%) had revascularization due to significantly larger CFC abnormalities, worse CFR, more clinical angina, and more angina or ST depression >1 mm during stress compared with severely reduced CFC with no revascularization (see Supplementary data online, *Table S9*).

## Discussion

### Role of comprehensive care for risk factor control to goals

The randomized CENTURY trial suggests that a comprehensive, integrated strategy of aggressive lifestyle modification and medical treatment towards risk factor goals with PET-derived CFC for safely limiting coronary interventions to severely compromised CFC significantly reduced cumulative risk factor scores, all-cause death, death or MI, and revascularization in chronic CAD patients. Review of risk factor data, PET images and frequent supportive participant contact with CENTURY research staff appeared to enhance adherence of the comprehensive compared with the standard care group (*Structured Graphical Abstract*).

### Role of positron emission tomography imaging

Experimental pathophysiology of coronary blood flow, coronary flow reserve (CFR), myocardial ischaemia and stenosis are well established^18–26^ and transitioned into the current knowledge base of clinical coronary pathophysiology.^7–16^ However, these basic pathophysiologic concepts and their transition into clinical application have not been validated by randomized trial of lifestyle, medical or invasive management of subclinical or manifest CAD. The randomized studies of FFR-guided PCI during invasive coronary angiography safely deferred or reduced subsequent need for revascularization in chronic CAD compared with angiographically guided PCI.^5,27^ Employing FFR for physiologic over angiographic severity for guiding PCI is an important conceptual and clinical advance as manifest in current ACC/AHA guidelines for interventions in CAD.^28^ However, the direct measurement of FFR requires invasive coronary angiography and intracoronary pressure measurements, does not reflect absolute stress perfusion or absolute CFR and, moreover, FFR-guided PCI did not reduce mortality.^5,27,28^

Quantitative myocardial perfusion by PET extended this basic experimental knowledge into clinical coronary pathophysiology including clinical FFR validated by PET stress perfusion,^29^ their combination as CFC,^7–10^ clinical autoregulation,^30^ the transmural perfusion gradient,^7,31,32^ coronary endothelial dysfunction,^33^ and microvascular dysfunction vs diffuse non-stenotic CAD.^7,34^ Thus, quantitative PET imaging offers assessment of physiologic significance of CAD for advancing beyond invasive pressure-based FFR to CFC per pixel as a comprehensive, artery-specific, size-severity physiologic threshold associated with mortality risk that is significantly improved after revascularization compared with no revascularization for similar severity in large non-randomized studies and this trial that is not provided by global perfusion metrics.^7–16^

For the population at high risk of CAD presenting in cardiology practice, this comprehensive care strategy provided objective reassurance to patients and physicians for safely pursuing lifestyle-medical treatment towards goals without the need for and at lower risk than invasive procedures for non-severely reduced CFC now commonly done. As gatekeeper for invasive coronary procedures, only 56 (5%) of 1028 participants had elective revascularization within 90 days after PET scans (*Table 3*) despite high prevalence of CAD manifest by coronary calcium, known or suspected chronic CAD, highly prevalent risk factors, symptoms, and abnormal PET as presenting to the practising cardiologists referring patients to this randomized trial.

Since quantitative perfusion metrics were unchanged from baseline to 5 years for either comprehensive care or standard care groups, the significantly reduced death, death or MI and revascularization procedures may be due to stabilization of atherosclerotic disease. These results suggest that prior randomized interventional trials may have demonstrated no survival benefit due to lack of physiologic severity inclusion criteria sufficient for survival benefit by revascularization or lack of sustained, frequent, long-term, supportive contacts with participants for achieving risk factor control to stringent goals.

### Role of revascularization

Revascularization in CENTURY was performed equally in each group for predominantly symptomatic, severely reduced CFC by PET having larger severe CFC regions, lower stress perfusion, lower CFR, more clinical angina and more angina or ST depression >1 mm during stress PET imaging than patients with severe CFC who were not revascularized (see Supplementary data online, *Table S4*). Death and death or MI over extended follow-up were higher for severe CFC, than for non-severe CFC (see Supplementary data online, *Table S8*). The differential beneficial outcomes for comprehensive over standard care group were the same after excluding revascularization from analysis. Residual or progressive CAD after revascularization with new or ongoing severely reduced CFC predicts ongoing high mortality risk that is significantly reduced by additional revscularization.^10^

### Comparison to the literature

The significantly improved hard outcomes of this randomized trial over extended follow-up provide definitive trial support of recent reviews on managing chronic CAD^35,36^ and 2024 ESC guidelines^36^ achieving substantially better risk factor goals than prior randomized trials.^1–5^ Our physiologic data suggesting plaque stabilization is consistent with small reduction in coronary percent atheroma volume (−2.02%) or increased minimum lumen area (0.21 mm) after vigorous LDL lowering by PCSK9 inhibition,^37^ and with favourable arterial remodelling.^38^ Finally, perfusion metrics of the CENTURY Trial using legacy 2D analogue PET-CT and Rb-82 has proven extendable to current digital 3D SiPM PET-CT for clinical decision making and further trials.^39^

### Limitations of the study

The randomized CENTURY trial was conducted at a large, single academic centre using quantitative PET myocardial perfusion. However, its scientific strengths include uniformity, consistency, data reproducibility derived from the same software, extensive scanner validations and calibrations over 15 years of the trial by the same experienced medical staff, physicians, PET treatment protocols, relational database, error scrubbing routines, PET-CT scanners, image acquisition, reconstruction software, and the same technologists. Although referral selection bias of not referring patients at high clinical risk cannot be ruled out, it did not preclude inclusion of substantial severity of CAD by PET imaging (*Table 1*). By direct or indirect patient contact (e.g. prescription refill), survival was proven (no death) in 874/1028 (85%) participants similarly for both groups (84% vs 86%). Of 1028 participants, 239/1028 (23%) withdrew from participating in either follow-up clinic visits or PET scans after their enrolment, similarly for both groups (23% and 24%) over extended follow-up for whom survival may have been ascertained.

Blinding coronary calcium on PET-CT in addition to myocardial perfusion images in the standard care group might be considered as a bias since CAC influences adherence. However, reviewing PET-CT images and CFC of our comprehensive integrated imaging-lifestyle-medical-interventional strategy was a protocol defined component for the comprehensive group intended to promote adherence vs standard community practice that includes widely available CAC. In our view, reporting CAC of PET-CT without perfusion for the standard group would violate our pre-defined protocol and likely lead to bias towards unnecessary angiograms and interventions in the standard group.

## Conclusions

The randomized, controlled, blinded 5-year CENTURY trial demonstrates that participants for whom invasive coronary procedures were safely deferred based on CFC by PET, integrated with comprehensive, intense lifestyle modifications and aggressive medical treatment targeted to goals significantly improved all risk factor scores with significant reduction in all-cause mortality by 42.7%, and death or MI by 37.0%, revascularization by 35.1% and MACE by 31.4% over extended follow-up compared with standard care in chronic CAD. These long-term beneficial hard outcomes of the CENTURY trial oriented to clinical practice warrant further research for potential wider application.

## Supplementary Material

ehaf356_Supplementary_Data

## Declarations

### Data Availability

If accepted for publication, de-identified data and statistical analysis will be made available on request.

### Pre-registered Clinical Trial Number

ClinicalTrials.gov NCT00756379.
