# Correction to: Optimal medical care and coronary flow capacity-guided myocardial revascularization vs usual care for chronic coronary artery disease: the CENTURY trial

**DOI:** 10.1093/eurheartj/ehaf446

**Published:** 2025-06-23

This is a correction to: K Lance Gould, Nils P Johnson, Amanda E Roby, Richard Kirkeeide, Mary Haynie, Tung Nguyen, Linh Bui, Monica B Patel, Danai Kitkungvan, Patricia Mendoza, Dejian Lai, Ruosha Li, Stefano Sdringola, David McPherson, Jagat Narula, Optimal medical care and coronary flow capacity-guided myocardial revascularization vs usual care for chronic coronary artery disease: the CENTURY trial, *European Heart Journal*, Volume 46, Issue 33, 1 September 2025, Pages 3273–3286, https://doi.org/10.1093/eurheartj/ehaf356

In the originally published version of this manuscript, in the Results section of the Abstract, an error was present in the following sentence, whereby “29.5%” should have been given as “20.5%” for the standard group:

Comprehensive vs standard care decreased risk factors and summed 5-year risk score (Δ−1.1 vs + 0.33; 95% confidence interval −1.84 to −0.97; *P* < .0001), decreased cumulative 11-year all-cause death (4.7% vs 8.2%; *P* = .023), death or MI (7.0% vs 11.1%; *P* = .024) late revascularization (9.5% vs 14.8%; *P* = .021) and major adverse cardiac events (29.5% vs 29.9%; *P* = .0006).

This has been corrected in the article and the revised sentence now reads as follows:

Comprehensive vs standard care decreased risk factors and summed 5-year risk score (Δ−1.1 vs + 0.33; 95% confidence interval −1.84 to −0.97; *P* < .0001), decreased cumulative 11-year all-cause death (4.7% vs 8.2%; *P* = .023), death or MI (7.0% vs 11.1%; *P* = .024) late revascularization (9.5% vs 14.8%; *P* = .021) and major adverse cardiac events (20.5% vs 29.9%; *P* = .0006).

