# Supplementary material for: Optimal medical care and coronary flow capacity-guided myocardial revascularization vs usual care for chronic coronary artery disease: the CENTURY trial
Source: Eur Heart J. 2025 May 29;46(33):3273–86. doi: 10.1093/eurheartj/ehaf356 (PMC12401582; doi:10.1093/eurheartj/ehaf356)

**Supplement** EURHEARTJ-D-24-03345 revised 3-28-25

**CONTENTS**

**Supplement tables S1-S9 are on supplement pages S1-S9.**

**Supplement figures S1 and S2 are on supplement pages S11-S13.**

**(Tables S1–S3 reproduced from reference 1).**

**Supplement Table S1. Inclusion and exclusion criteria.^1^**

| **Inclusion criteria** | **Exclusion criteria** |
| --- | --- |
| - Men and women aged ≥40 years - Established CAD as documented by abnormal myocardial perfusion imaging, CT angiography, invasive coronary angiography, or previous revascularization procedures **OR** - Framingham cardiovascular event risk ≥10% at 10 years in the absence of established CAD at study entry - Subclinical or suspected stable CAD criteria including symptoms or other signs such as CAC score, high risk factors for CAD, poor ventricular function or referral to a PET-CT scan for clinical question of CAD, microvascular disease, heart failure, past history of CAD, MI or abnormal angiogram, family history of CAD. | - Age <40 years - Morbid obesity, defined as BMI >35 kg/m^2^ - Low pretest likelihood of CAD (all categories not meeting those Inclusion Criteria listed on the left). - High risk unstable angina (dynamic ST–T ECG changes, elevated troponin level or both) - Recent (<4 weeks) myocardial Infarction - Recent (<4 weeks) stroke - CABG surgery or PCI within the past 6 months - Significant renal dysfunction, defined as creatinine level >2.0 mg/dl - Active liver disease or hepatic dysfunction (ALT or AST >x 3 the upper limit of normal) - Concomitant severe valvular heart disease - Congenital or primary cardiomyopathy likely to affect prognosis during follow-up - LVEF <30% - Severe sustained systemic hypertension, defined as documented SBP >200 mmHg - Symptomatic sustained or non-sustained ventricular tachycardia - Sufficient disability to prevent therapeutic exercise not expected to resolve within 6 months - Major non-cardiac co-morbidity limiting survival or social situation/condition that, in the opinion of the investigator, preclude the patient from participation in study follow-up - Concurrent or prior (within past 30 days) participation in other research studies using investigational drugs or devices |

ALT= alanine transaminase, AST= aspartate transaminase, BMI=body mass index, CABG=coronary artery bypass graft, CAD=coronary artery disease, CT=computed tomography, ECG=electrocardiographic, LVEF=left ventricular ejection fraction, PCI=percutaneous coronary intervention, SBP, systolic blood pressure.

**Supplement Table S2. Risk score components.** All modifiable risk factors over the widest range of continuous increasing risk (LDL, HBAic, smoking, age) are scored 0 (none) to 4 (maximal risk). Other risk factors with a narrower range of progressive risk for increasing (HDL, triglycerides, blood pressure, BMI, and inversely for exercise METs, diet, and medication adherence) are scored 0 (none) to 3 (maximum risk). Fixed risk factors have a single fixed score. “Not used” indicates that the zero to maximum risk was accounted for by the assigned risk score range without need higher risk scores for that risk factor. Risk scores were assigned for each patient at each visit by an experienced team of blinded cardiologist, nurse, nutritionist and research assistants.

|  |  | | | | |
| --- | --- | --- | --- | --- | --- |
|  | **0** | **1** | **2** | **3** | **4** |
| **Modifiable risk factors** | | | | | |
| **LDL (mg/dl)** | **<70** | **70–99** | **100–129** | **130–159** | **≥160** |
| **HDL (mg/dl)** | **≥45** | **35–44** | **<35** | **Not used** | **Not used** |
| **Triglycerides (mg/dl)** | **<100** | **100-149** | **≥150** | **Not used** | **Not used** |
| **HBA_1C_ (%)** | **<5.5** | **5.5–5.9** | **6.0–7.4** | **7.5–8.9** | **≥9.0** |
| **Blood pressure (mmHg)** | **< 135/80** | **135–144/80–89** | **≥145/90** | **Not used** | **Not used** |
| **BMI (kg/m^2^)** | **<25** | **25–29** | **≥30** | **Not used** | **Not used** |
| **METS on ETT*** | **≥10** | **5–10** | **<5** | **Not used** | **Not used** |
| **Diet score** | **9–12** | **5–8** | **<5** | **Not used** | **Not used** |
| **Smoking** | **none** | **≤10 cigarette/week** | **<10 cigarette/day** | **½ -1 pack/day** | **>1 pack/day** |
| **Adherence to medications** | **<50%** | **50–80%** | **>80%** | **Not used** | **Not used** |
| **Non-modifiable risk factors** | | | | | |
| **Age (years)** | **<50** | **50–54** | **55–64** | **65–69** | **≥70** |
| **Gender** | **Female** | **Male or postmenopausal Female** | **Not used** | **Not used** | **Not used** |
| **Family history**** | **None** | **Not used** | **In Parent or sibling** | **Not used** | **Not used** |
| **Diabetes mellitus (separate from HbA_1C_)** | **Absent** | **Present** | **Not used** | **Not used** | **Not used** |
| **CAC >120*** Hounsfield units** | **Absent** | **Present** | **Not used** | **Not used** | **Not used** |
| **Established CAD** | **Absent** | **Not used** | **Present** | **Not used** | **Not used** |

*Treadmill stress test Bruce protocol. **Premature vascular disease at age <60 years. ***On CT for PET attenuation correction. BMI=body mass index, CAC=coronary artery calcium, CAD, coronary artery disease, CT=computed tomography, ETT=exercise tolerance testing, HBA1C=haemoglobin A_1C,_ HDL=high-density lipoprotein, LDL=low-density lipoprotein, METS=metabolic equivalents, PET=positron emission tomography.

**Supplement Table S3.** **Dietary score with 12 components determined personalized for each participant at each clinic visit.^1^**

| **Criteria** | **Dietary score*** | |
| --- | --- | --- |
| **Threshold for each of 12 food items** | **0** | **1** |
| Total calories per day individualized | Above target | Below target |
| Total fat ≥ 20 g/day | Yes | No |
| ≥10% of total calories derived from fat | Yes | No |
| Saturated fat ≥7% of total fat | Yes | No |
| Cholesterol ≥100 mg/day | Yes | No |
| Carbohydrates > personal target g/day | Yes | No |
| Sodium ≥ 2.4 g/day | Yes | No |
| Nuts <2 servings per week | No | Yes |
| Protein ≥ 60 g/day | No | Yes |
| Dietary fibre >25 g/day | No | Yes |
| Vegetables and fruits ≥5 servings per day | No | Yes |
| Fish ≥2 servings per week | No | Yes |

*Worst (unhealthy) score = 0. Best (healthiest score) = 12.

**Supplement Table S4. Schedule for clinic visits and testing for study patients.^1^**

|  | **Standard group** | | | | | |  | **Comprehensive group** | | | | | | | | | | | | | |
| --- | --- | --- | --- | --- | --- | --- | --- | --- | --- | --- | --- | --- | --- | --- | --- | --- | --- | --- | --- | --- | --- |
| **Test** | **Month** | | | | | |  | **Month** | | | | | | | | | | | | | |
|  | **0** | **12** | **24** | **36** | **48** | **60** |  | **0** | **1** | **2** | **4** | **8** | **12** | **18** | **24** | **30** | **36** | **42** | **48** | **54** | **60** |
| Clinical history | x | x | x | x | x | x |  | x | x | x | x | x | x | x | x | x | x | x | x | x | x |
| Physical examination | x | x | x | x | x | x |  | x | x | x | x | x | x | x | x | x | x | x | x | x | x |
| Blood test* | x |  | x |  |  | x |  | x |  |  |  |  |  |  | x |  |  |  |  |  | x |
| Physical activity assessment | x | x | x | x | x | x |  | x | x | x | x | x | x | x | x | x | x | x | x | x | x |
| Exercise stress test | x |  | x |  |  | x |  | x |  |  |  |  | x |  | x |  | x |  | x |  | x |
| Dietary assessment | x | x | x | x | x | x |  | x | x | x | x | x | x | x | x | x | x | x | x | x | x |
| Quality of life assessment | x | x | x | x | x | x |  | x | x | x | x | x | x | x | x | x | x | x | x | x | x |
| Diet and exercise consultation |  |  |  |  |  |  |  | x | x | x | x | x | x | x | x | x | x | x | x | x | x |
| Physician consultation |  |  |  |  |  |  |  | x | x | x | x | x | x | x | x | x | x | x | x | x | x |
| PET scan | x |  | x |  |  | x |  | x |  |  |  |  |  |  | x |  |  |  |  |  | x |

*Includes fasting lipid profile, haemoglobin A_1C_ and comprehensive metabolic panel. PET=positron emission tomography.

**Supplement Table S5. Comparison of two sample t test with mixed linear model analysis of risk score changes**

Baseline risk scores for comprehensive-care and standard-care groups are comparable (see figure S1 below). Final 5-year risk score significantly

decreases to a lower risk in the comprehensive-care group compared with a significant increase to a higher risk in the standard-care group. Results

from two sample t test and linear mixed model are very similar to each other

| **Analysis Method** |  | **Mean (95% CI)** | **t-value** | **p-value** |
| --- | --- | --- | --- | --- |
| **Two-sample t-test** | Baseline risk: comprehensive care vs standard care | 0.17 (-0.25, 0.59) | 0.79 | 0.43 |
|  | Final risk: comprehensive care vs standard care | -1.23 (-1.72, -0.74) | -4.95 | <.0001 |
|  | Change in risk: comprehensive care vs standard care | -1.40 (-1.84, -0.97) | -6.36 | <.0001 |
| **Linear mixed model** | Baseline risk: comprehensive care vs standard care | 0.16 (-0.26, 0.57) | 0.73 | 0.47 |
|  | Final risk: comprehensive care vs standard care | -1.32 (-1.77, -0.87) | -5.78 | <.0001 |
|  | Change in risk: comprehensive care vs standard care | -1.48 (-1.84, -1.11) | -7.99 | <.0001 |

Protocol deviations consisted primarily of patients changing or missing follow up clinic appointments that reduced the number of completed 0 to 5-year risk scores as indicated in supplement table S6a. For a sensitivity analysis taking into account incomplete risk scores, a linear mixed model with subject-level random intercept was also used to model the summed risk score at baseline and yearly, follow-up visits including time (0[reference],2 2. 3. 4 and 5 years), randomization group, and group by time interaction as fixed effects. Of main interest is the coefficient of the interaction term between groups and 5-year risk acores, which corresponds to the between-group mean difference in the ∆ summed risk score from baseline to 5-year (supplement table S6b).

**Supplement Table S6. Histogram distribution of baseline to 5 year change in risk score (∆)**

| **∆ Comprehensive-care risk score** | |  | **∆ Standard-care risk score** | |  |
| --- | --- | --- | --- | --- | --- |
| **Change in risk score** | **Frequency** | **Cumulative %** | **Change in risk score** | **Frequency** | **Cumulative %** |
| -14 | 0 | 0.00% | -14 | 0 | 0.00% |
| -13 | 0 | 0.00% | -13 | 0 | 0.00% |
| -12 | 0 | 0.00% | -12 | 1 | 0.27% |
| -11 | 0 | 0.00% | -11 | 0 | 0.27% |
| -10 | 0 | 0.00% | -10 | 0 | 0.27% |
| -9 | 3 | 0.78% | -9 | 0 | 0.27% |
| -8 | 6 | 2.33% | -8 | 1 | 0.53% |
| -7 | 10 | 4.91% | -7 | 2 | 1.07% |
| -6 | 10 | 7.49% | -6 | 5 | 2.40% |
| -5 | 16 | 11.63% | -5 | 9 | 4.80% |
| -4 | 27 | 18.60% | -4 | 20 | 10.13% |
| -3 | 45 | 30.23% | -3 | 25 | 16.80% |
| -2 | 54 | 44.19% | -2 | 29 | 24.53% |
| -1 | 59 | 59.43% | -1 | 49 | 37.60% |
| **0** | **42** | **70.28%** | **0** | **57** | **52.80%** |
| 1 | 47 | 82.43% | 1 | 59 | 68.53% |
| 2 | 24 | 88.63% | 2 | 43 | 80.00% |
| 3 | 17 | 93.02% | 3 | 30 | 88.00% |
| 4 | 9 | 95.35% | 4 | 15 | 92.00% |
| 5 | 12 | 98.45% | 5 | 12 | 95.20% |
| 6 | 5 | 99.74% | 6 | 7 | 97.07% |
| 7 | 1 | 100.00% | 7 | 1 | 97.33% |
| 8 | 0 | 100.00% | 8 | 4 | 98.40% |
| 8 | 0 | 100.00% | 8 | 0 | 98.40% |
| 9 | 0 | 100.00% | 9 | 2 | 98.93% |
| 9 | 0 | 100.00% | 9 | 0 | 98.93% |
| 10 | 0 | 100.00% | 10 | 4 | 100.00% |
| 10 | 0 | 100.00% | 10 | 0 | 100.00% |
| 11 | 0 | 100.00% | 11 | 0 | 100.00% |
| 11 | 0 | 100.00% | 11 | 0 | 100.00% |
| 12 | 0 | 100.00% | 12 | 0 | 100.00% |
| 12 | 0 | 100.00% | 12 | 0 | 100.00% |
| 13 | 0 | 100.00% | 13 | 0 | 100.00% |
| 14 | 0 | 100.00% | 14 | 0 | 100.00% |
| More | 0 | 100.00% | More | 0 | 100.00% |

**Supplement Table S7. For all patients with CFC severe PETs: comprehensive vs standard group.**

| **Characteristic** | **Overall, N = 243^1^** | **Comprehen, N = 126^1^** | **Standard, N = 117^1^** | **p value^2^** |
| --- | --- | --- | --- | --- |
| **Age** | **67.53 (9.83)** | **67.76 (9.76)** | **67.29 (9.95)** | **0.7** |
| **Male** | **205 / 243 (84%)** | **105 / 126 (83%)** | **100 / 117 (85%)** | **0.6** |
| **BMI** | **29.10 (4.02)** | **28.99 (4.20)** | **29.22 (3.82)** | **0.7** |
| **Diabetes** | **183 / 243 (75%)** | **98 / 126 (78%)** | **85 / 117 (73%)** | **0.4** |
| **Hypertension** | **220 / 243 (91%)** | **118 / 126 (94%)** | **102 / 117 (87%)** | **0.085** |
| **Dyslipidemia** | **239 / 243 (98%)** | **122 / 126 (97%)** | **117 / 117 (100%)** | **0.12** |
| **Coronary Calcium** | **239 / 243 (98%)** | **123 / 126 (98%)** | **116 / 117 (99%)** | **0.6** |
| **History of MI** | **78 / 243 (32%)** | **40 / 126 (32%)** | **38 / 117 (32%)** | **>0.9** |
| **Prior Revascularizatn** | **142 / 243 (58%)** | **74 / 126 (59%)** | **68 / 117 (58%)** | **>0.9** |
| **Clin Angina Typ/Atyp** | **44 / 243 (18%)** | **21 / 126 (17%)** | **23 / 117 (20%)** | **0.5** |
| **Typical clinical angina** | **36 / 243 (15%)** | **15 / 126 (12%)** | **21 / 117 (18%)** | **0.2** |
| **Ejection Fraction** | **62.90 (10.54)** | **63.19 (10.22)** | **62.58 (10.91)** | **0.7** |
| **PET Perfusion Defect** | **0.10 (0.13)** | **0.10 (0.12)** | **0.10 (0.14)** | **>0.9** |
| **Absolute Stress Flow** | **1.41 (0.42)** | **1.44 (0.40)** | **1.37 (0.44)** | **0.2** |
| **Absolute CFR** | **1.84 (0.42)** | **1.82 (0.42)** | **1.86 (0.41)** | **0.5** |
| **Rest MQA^3^** | **0.69 (0.22)** | **0.72 (0.24)** | **0.66 (0.20)** | **0.04** |
| **Stress MQA^3^** | **1.12 (0.39)** | **1.14 (0.39)** | **1.09 (0.39)** | **0.3** |
| **CFR MQA^3^** | **1.55 (0.40)** | **1.54 (0.43)** | **1.56 (0.37)** | **0.7** |
| **PET angina** | **45 / 243 (19%)** | **21 / 126 (17%)** | **24 / 117 (21%)** | **0.4** |
| **PET ST > 1mm** | **58 / 243 (24%)** | **28 / 126 (22%)** | **30 / 117 (26%)** | **0.5** |
| **PET angina-ST>1mm** | **74 / 243 (30%)** | **34 / 126 (27%)** | **40 / 117 (34%)** | **0.2** |
| **CFC severe % of LV** | **0.08 (0.12)** | **0.07 (0.10)** | **0.09 (0.13)** | **0.4** |
| **CFC moderate % of LV** | **0.13 (0.13)** | **0.13 (0.12)** | **0.14 (0.14)** | **0.4** |
| **CFC mild % of LV** | **0.48 (0.23)** | **0.49 (0.22)** | **0.47 (0.24)** | **0.7** |
| **CFC minimal % of LV** | **0.16 (0.14)** | **0.17 (0.15)** | **0.15 (0.14)** | **0.3** |
| **CFC normal % of LV** | **0.12 (0.18)** | **0.12 (0.18)** | **0.12 (0.18)** | **0.9** |
| **CFCsevere # (%)** | **243 / 243 (100%)** | **126 / 126 (100%)** | **117 / 117 (100%)** |  |
| **Revasc < 90d** | **46 / 243 (19%)** | **23 / 126 (18%)** | **23 / 117 (20%)** | **0.8** |
| **Revasc post PET** | **53 / 243 (22%)** | **25 / 126 (20%)** | **28 / 117 (24%)** | **0.4** |

^1^ Mean (SD). ^2^ Welch 2 Sample t-test; Pearson’s Chi-sq test; Fisher’s exact test.^3^ MQA minimum quadrant avg

**Supplement Table S8. Characteristics of 56 participants with revascularization at ≤ 90days vs no revascularization of which 46 had severely reduced CFC.**

| **Characteristic** | **Overall** | **No revasc ≤90d** | **Revasc ≤90d** | **p-value^2^** | ***Revasc ≤90d is / has*** |
| --- | --- | --- | --- | --- | --- |
| **CENTURY Arm** | **N = 1,028^1^** | **N = 971^1^** | **N =56 (5.4%)^1^** |  |  |
| **Comprehensive** | **513 / 1,027 (50%)** | **487 / 971 (50%)** | **26 / 56 (46%)** | **0.6** |  |
| **Standard** | **514 / 1,027 (50%)** | **484 / 971 (50%)** | **30 / 56 (54%)** |  |  |
| **Age** | **62.12 (9.14)** | **61.91 (9.05)** | **65.79 (10.01)** | **0.006** | ***older*** |
| **Male** | **696 / 1,027 (68%)** | **648 / 971 (67%)** | **48 / 56 (86%)** | **0.003** | ***more male*** |
| **BMI** | **28.84 (3.81)** | **28.85 (3.80)** | **28.67 (4.12)** | **0.8** |  |
| **Diabetes** | **675 / 1,027 (66%)** | **640 / 971 (66%)** | **35 / 56 (63%)** | **0.6** |  |
| **Hypertension** | **868 / 1,027 (85%)** | **816 / 971 (84%)** | **52 / 56 (93%)** | **0.076** |  |
| **Dyslipidemia** | **1,015 / 1,027 (99%)** | **959 / 971 (99%)** | **56 / 56 (100%)** | **>0.9** |  |
| **Coronary Calcium** | **919 / 1,027 (89%)** | **863 / 971 (89%)** | **56 / 56 (100%)** | **0.008** | ***both with cor calci*** |
| **History of MI** | **176 / 1,027 (17%)** | **159 / 971 (16%)** | **17 / 56 (30%)** | **0.007** | ***more clinical CAD*** |
| **Prior Revascularization** | **343 / 1,027 (33%)** | **310 / 971 (32%)** | **33 / 56 (59%)** | **<0.001** | ***more prior revasc*** |
| **Clinical Angina Typ/Atyp** | **115 / 1,027 (11%)** | **93 / 971 (9.6%)** | **22 / 56 (39%)** | **<0.001** | ***more chest pain*** |
| **Typical clinical angina** | **75 / 1,027 (7.3%)** | **58 / 971 (6.0%)** | **17 / 56 (30%)** | **<0.001** | ***more angina*** |
| **Ejection Fraction** | **68.05 (9.41)** | **68.50 (9.09)** | **60.31 (11.50)** | **<0.001** | ***both with good EF*** |
| **PET Perfusion Defect** | **0.02 (0.08)** | **0.01 (0.06)** | **0.20 (0.17)** | **<0.001** | ***larger rel PET defect*** |
| **Absolute Stress Flow** | **2.02 (0.65)** | **2.05 (0.64)** | **1.43 (0.51)** | **<0.001** | ***worse stress flow*** |
| **Absolute CFR** | **2.49 (0.65)** | **2.52 (0.63)** | **1.94 (0.67)** | **<0.001** | ***lower global CFR*** |
| **Rest Min Quad Avg** | **0.76 (0.26)** | **0.76 (0.26)** | **0.70 (0.29)** | **0.12** |  |
| **Stress Min Quad Avg** | **1.81 (0.66)** | **1.85 (0.64)** | **1.03 (0.43)** | **<0.001** | ***lower avg quad flow*** |
| **CFR Min Quad Avg** | **2.28 (0.65)** | **2.32 (0.62)** | **1.48 (0.55)** | **<0.001** | ***lower quad CFR*** |
| **PET angina** | **62 / 1,027 (6.0%)** | **36 / 971 (3.7%)** | **26 / 56 (46%)** | **<0.001** | ***more PET symptoms*** |
| **PET ST∆ > 1mm** | **93 / 1,027 (9.1%)** | **64 / 971 (6.6%)** | **29 / 56 (52%)** | **<0.001** | ***more ST depression*** |
| **PET angina or ST>1mm** | **122 / 1,027 (12%)** | **85 / 971 (8.8%)** | **37 / 56 (66%)** | **<0.001** | ***more angina or ST∆*** |
| **CFC severe % of LV** | **0.02 (0.07)** | **0.01 (0.05)** | **0.14 (0.16)** | **<0.001** | ***larger % LV sev CFC*** |
| **CFC moderate % of LV** | **0.03 (0.09)** | **0.03 (0.08)** | **0.12 (0.10)** | **<0.001** | ***larger %LV mod CFC*** |
| **CFC mild % of LV** | **0.23 (0.27)** | **0.22 (0.27)** | **0.35 (0.19)** | **<0.001** | ***larger %LV mildCFC*** |
| **CFC minimal % of LV** | **0.22 (0.18)** | **0.23 (0.18)** | **0.17 (0.11)** | **<0.001** | ***smaller%LV minCFC*** |
| **CFC normal % of LV** | **0.48 (0.37)** | **0.50 (0.37)** | **0.20 (0.25)** | **<0.001** | ***smaller%LV normal*** |
| **CFC severely reduced #** | **243 / 1,027 (24%)** | **196 / 971 (20%)** | **46 / 56 (82%)** | **<0.001** | ***more % pts sev CFC*** |

^1^ n / N (%); Mean (SD).

^2^ Pearson’s Chi-squared test; Welch Two Sample t-test; Fisher’s exact test

^3^ Over extended follow-up to 11 years

^* revascularization at > 90days^

**Analysis of median (lower-upper quartiles) by Wolcoxon P values showed no difference in significance from the P values in the above table.**

**Supplement Table S9. Demographic characteristics of participants with revascularization vs no revascularisation for 46 with severely reduced CFC.**

| **Comprehen +Standard**  **total N = 1028 CENTURY** | **Overall CFCsev**  **N = 243*** | **No revascularis**  **N = 197*** | **Revasc<90d N = 46*** | **p-value†** |
| --- | --- | --- | --- | --- |
| **CENTURY with severe CFC** | **243//1028 (24%)** | **197/243 (81%)** | **46/242 (19%)** |  |
| **Comprehensive** | **126 / 243 (52%)** | **103 / 197 (53%)** | **23 / 46 (50%)** | **0.8** |
| **Standard** | **117 / 243 (48%)** | **94 / 197 (47%)** | **23 / 46 (50%)** |  |
| **Age** | **67.6 (9.9)** | **67.9 (9.8)** | **66.1 (9.9)** | **0.3** |
| **Male** | **205 / 243 (84%)** | **166 / 197 (84%)** | **39 / 46 (85%)** | **>0.9** |
| **BMI** | **29.1 (4.0)** | **29.1 (4.0)** | **29.0 (4.3)** | **>0.9** |
| **Diabetes** | **183 / 243 (75%)** | **155 / 197 (79%)** | **28 / 46 (61%)** | **0.012** |
| **Hypertension** | **220 / 243 (90%)** | **177 / 197 (90%)** | **43 / 46 (93%)** | **0.6** |
| **Dyslipidemia** | **239 / 243 (98%)** | **193 / 197 (98%)** | **46 / 46 (100%)** | **>0.9** |
| **Coronary Calcium** | **239 / 243 (98%)** | **193 / 197 (98%)** | **46 / 46 (100%)** | **>0.9** |
| **History of MI** | **78 / 243 (32%)** | **63 / 197 (32%)** | **15 / 46 (33%)** | **>0.9** |
| **Prior Revascularization** | **142 / 243 (58%)** | **116 / 197 (59%)** | **26 / 46 (57%)** | **0.8** |
| **Clinical AnginaTyp/Atyp** | **44 / 243 (18%)** | **25 / 197 (12%)** | **19 / 46 (41%)** | **<0.001** |
| **Typical clinical angina** | **36 / 243 (14%)** | **21 / 197 (10%)** | **15 / 46 (33%)** | **<0.001** |
| **Ejection Fraction** | **62.9 (10.5)** | **63.6 (10.3)** | **60.1 (11.1)** | **0.066** |
| **PET Perfusion Defect** | **0.10 (0.13)** | **0.07 (0.10)** | **0.23 (0.17)** | **<0.001** |
| **Absolute Stress Flow** | **1.41 (0.43)** | **1.43 (0.41)** | **1.35 (0.49)** | **0.3** |
| **Absolute CFR** | **1.84 (0.42)** | **1.85 (0.40)** | **1.77 (0.49)** | **0.3** |
| **Rest Min Quadrant Avg** | **0.69 (0.22)** | **0.69 (0.2)** | **0.72 (0.30)** | **0.5** |
| **Stress Min Quadrant Avg** | **1.12 (0.39)** | **1.16 (0.38)** | **0.94 (0.38)** | **<0.001** |
| **CFR Min Quadrant Avg** | **1.55 (0.40)** | **1.60 (0.37)** | **1.32 (0.44)** | **<0.001** |
| **PET angina** | **45 / 243 (19%)** | **23 / 197 (12%)** | **22 / 46 (48%)** | **<0.001** |
| **PET ST changes > 1mm** | **58 / 243 (24%)** | **32 / 197 (16%)** | **26 / 46 (57%)** | **<0.001** |
| **PET angina or ST>1mm** | **74 / 243 (30%)** | **43 / 197 (22%)** | **31 / 46 (67%)** | **<0.001** |
| **CFC severe % of LV** | **0.08 (0.12)** | **0.06 (0.09)** | **0.17 (0.16)** | **<0.001** |
| **CFC moderate % of LV** | **0.13 (0.13)** | **0.13 (0.14)** | **0.14 (0.10)** | **0.9** |
| **CFC mild % of LV** | **0.48 (0.23)** | **0.50 (0.23)** | **0.38 (0.19)** | **<0.001** |
| **CFC minimal % of LV** | **0.16 (0.14)** | **0.16 (0.15)** | **0.14 (0.10)** | **0.3** |
| **CFC normal % of LV** | **0.12 (0.18)** | **0.11 (0.18)** | **0.14 (0.21)** | **0.4** |
| **CFC severely reduce # (%)** | **243 / 243 (100%)** | **197 / 197 (100%)** | **46 / 46 (100%)** |  |
| **Revascularized post PET**** | **53 / 243 (22%)** | **7 / 197 (3.6%)** | **46 / 46 (100%)** | **<0.001** |
| **Death or MI**** | **55 / 243 (23%)** | **41 / 197 (21%)** | **14 / 46 (30%)** | **0.2** |
| **Death**** | **36 / 243 (15%)** | **28 / 197 (14%)** | **8 / 46 (17%)** | **0.6** |

* n / N (%) † Mean (SD) Pearson’s Chi-squared test. Welch Two Sample t-test. Fisher’s exact

test ** Over extended follow-up to 11 years.

**Analysis of median (lower-upper quartiles) by Wolcoxon P values showed no difference in significance from the P values in the above table.** **Supplement figure S1. Coronary flow capacity (CFC) map by positron emission tomography.**


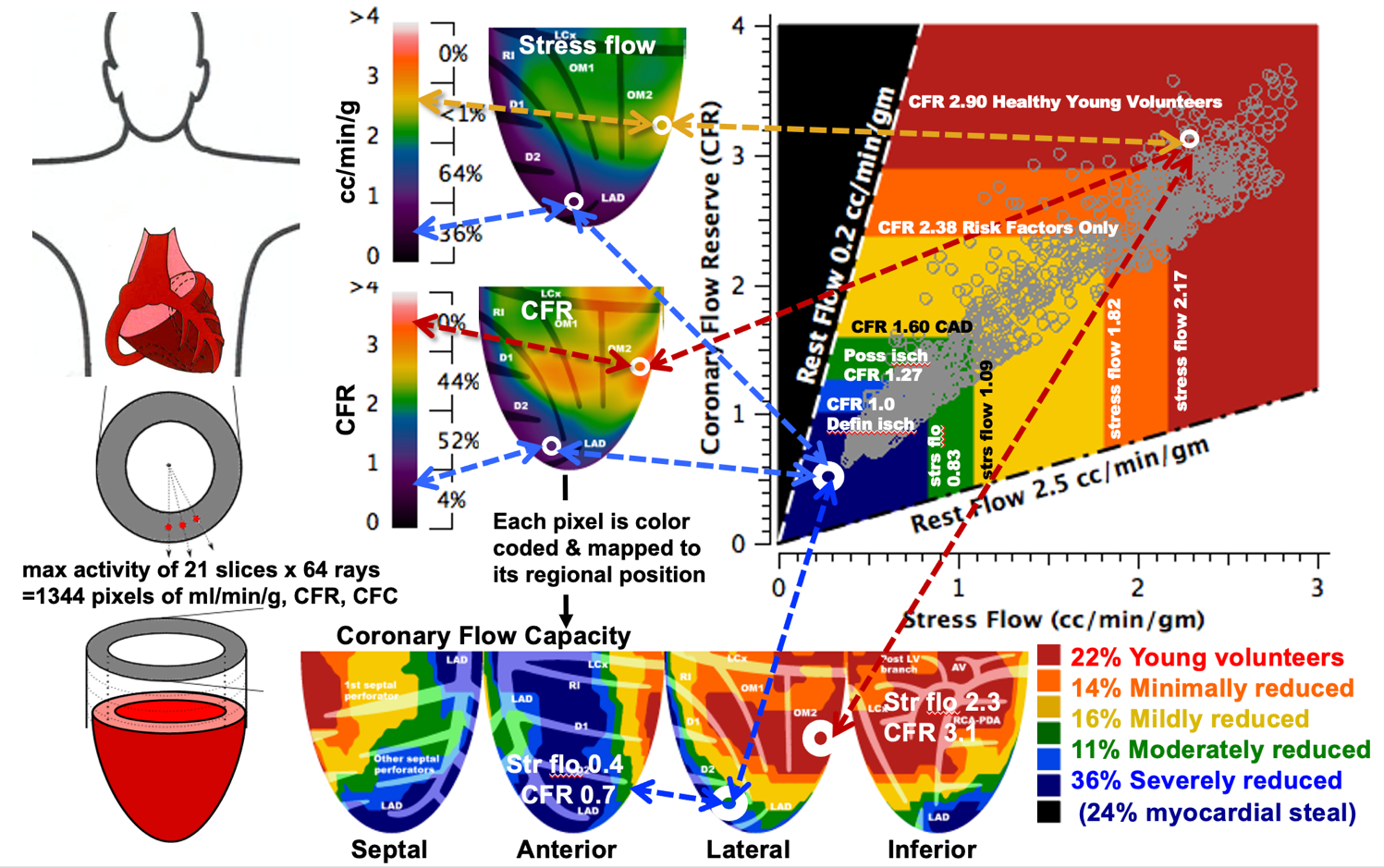


Positron emission tomography (PET) quantitative rest–stress ml/min/g per regional pixel of the left ventricle (LV) integrated into the comprehensive coronary flow capacity (CFC) map for abnormalities of each coronary artery down to tertiary branches. Regional per pixel quantitative perfusion avoids the errors incurred by arbitrary, externally imposed regions of interest with overlapping arterial distribution that distort size/severity perfusion quantification for specific arteries and branches.

Regional CFC based on pixel values of coronary flow reserve (CFR) and stress (cc/min/g). The wide range of stress perfusion in ml/min/g and CFR values for each of 1344 radial pixels comprise vast numbers of possible stress perfusion and CFR pixel combinations that are compressed into the following objectively determined ranges of combined values for each regional pixel by receiver operating characteristic curve (ROC) analysis as follows: excellent (CFR >2.9 and stress perfusion >2.17 cc/min/g, red), typical (CFR >2.38 to 2.9 and stress perfusion >1.82 to 2.17, orange), mildly reduced but not ischaemic (CFR >1.6 to 2.38 and stress perfusion >1.09 to 1.82, yellow), moderately reduced (CFR >1.27 to 1.6 and stress perfusion >0.83 to 1.09, green), severely reduced (CFR 1.0 to 1.27 and stress perfusion ≤0.83, blue), and myocardial steal (CFR <1.0, purple).

Each colour-coded pixel is spatially mapped back onto its LV location with percentage of LV calculated for each range of combined both CFR and stress perfusion pixel values listed in the CFC colour histogram bar. The regional, colour-coded 1344 pixels provide integrated, size/severity quantification for a specific coronary artery distribution down to tertiary branches as detailed in the text (FDA K202679).

**Healthy young volunteers (n = 212); red**

Healthy young volunteers aged <40 years with no vascular risk factors, normal measured lipid profile, no obesity, no measurable caffeine on blood samples for every PET, and no blood or urine cotinine levels measured for every PET.

**Risk factors only (n = 2171); orange**

Subjects with one or more vascular risk factors, but no known coronary artery disease (CAD) pre-PET as defined by no history of myocardial infarction (MI) percutaneous coronary intervention (PCI), coronary artery bypass graft (CABG) surgery, abnormal coronary angiogram, or angina, or presence of these factors at PET, no dipyridamole stress-induced ischaemia, and no myocardial scar (both as defined below).

**Established CAD (n = 979); yellow**

Patients with CAD defined by pre-PET history of MI, PCI, CABG surgery, or abnormal coronary angiogram. Patients with definite or possible ischaemia during dipyridamole stress (as defined below) were excluded from this group because they are classified in the next two groups. Patients with significant myocardial scar (as defined below) were also excluded to avoid the downward bias in thresholds of perfusion and CFR due to low flow of scars unrelated to stress.

**Possible ischaemia during dipyridamole stress PET (n =548) green**

Patients defined as having possible ischaemia requiring any one of the three following criteria during dipyridamole stress PET:

(i) A significant perfusion defect on stress images with >5% rest-to-stress change in % of LV with ≤ 60% maximum on relative uptake stress images that is > 3 standard devations (SD) below the mean of rest and stress relative PET images of healthy young volunteers, or

(ii) ST-segment depression >1mm on an electrocardiogram (ECG) that was not present on resting ECG, or

(iii) Definite angina requiring reversal by aminophylline, nitroglycerin, or intravenous metoprolol.

**Definite ischemia during dipyridamole stress PET (n = 278); blue**

Patients with definite ischaemia defined as having:

(i) A significant perfusion defect on stress images with >5% rest-to-stress change in % of LV with ≤ 60%

maximum on relative uptake stress images that is >3 SD below the mean of rest and stress relative PET images of healthy young volunteers, plus one or both of the following criteria:

(ii) ST-segment depression >1mm on ECG that was not present on resting ECG, or

(iii) Definite angina requiring reversal by aminophylline, nitroglycerin or intravenous metoprolol (as we have previously published with area under the curve (AUC) of 0.97).

Patients with myocardial scar were excluded by definition in the colour-coded CFC severity ranges above, so that the thresholds for stress flow and CFR would reflect the true stress perfusion and CFR changes without the downward bias due to low flow of scars unrelated to stress. The perfusion boundaries between clinical groups were determined by objective ROC analysis for optimum CFR and stress perfusion separating the predefined clinical groups and colour coded for the thresholds listed above.

**PET report interpretation for Supplement Figure S1**

Page 11 lines9-26, page 12 lines 1-26, page 13 lines 1-5 and page 17 line11-26 and page 18 line 1-18 summarized PET severity and reporting. Additional detail are inserted into the Supplement page 10 at the endo of the Legend Supplement Figure S1. As we have previously published, the CFC size-severity is a continuum with an associated continuum of size related risk quantified objectively in 12000 cases by Cox regression modeling of 10-year survival probability as a fraction of I.0. This probability is augmented in our PET reports as no, low, moderate or high probability of survival benefit related to size, distal or proximal stress defects, diffuse CAD, microvascular disease, low EF out of proportion to quantitative objective size of transmural and subendocardial scar indicating an component of non-ischemic cardiomyopathy. Each report also incorporates our integrated clinical judgement considering age, gender, symptoms, risk factors, prior procedures, prior angiograms, with a recommendation for, against or either medical Rx or intervention, qualified by the statement "Depending on clinical judgement, PET findings suggest the following:=== . This careful wording has been necessary to make data driven suggestions to invasive cardiologist who resist anyone "telling" them what to or feeling "trapped" by a strong PET recommendation against procedures that PET suggest have no or low probability of benefiting EF, survival or symptoms, particularly in the absence of definite angina.

**Supplement Figure S2 Risk Factor Score at baseline and 5 years**

**
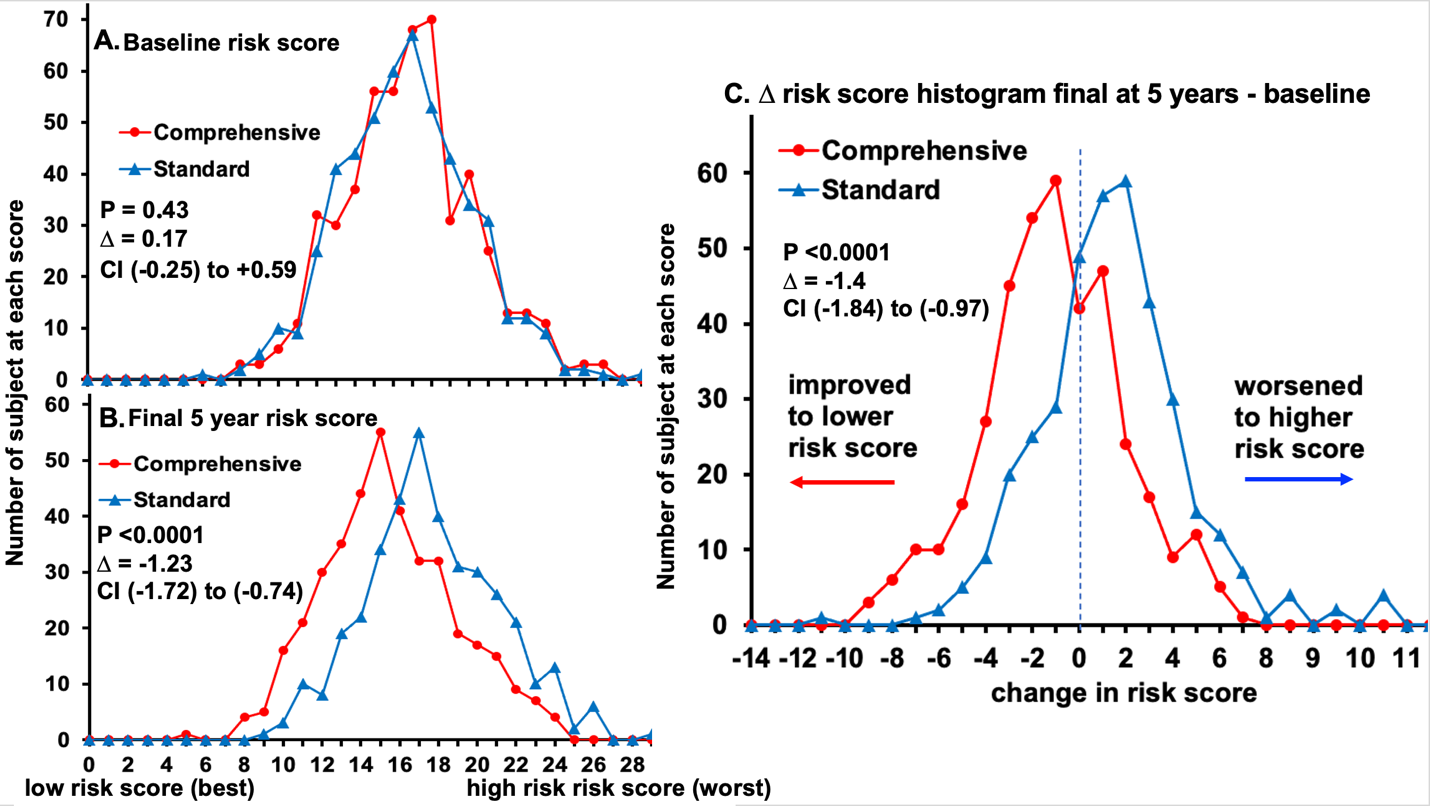
**

**Supplement References**

S1. Kitkungvan D, Johnson NP, Kirkeeide R, et al. Design and rationale of the randomized trial of comprehensive lifestyle modification, optimal pharmacological treatment and utilizing PET imaging for quantifying and managing stable coronary artery disease (the CENTURY study). *Am Heart J* 2021; **237:** 135–46.

**Supplement Figure S3 Cox regression analysis**


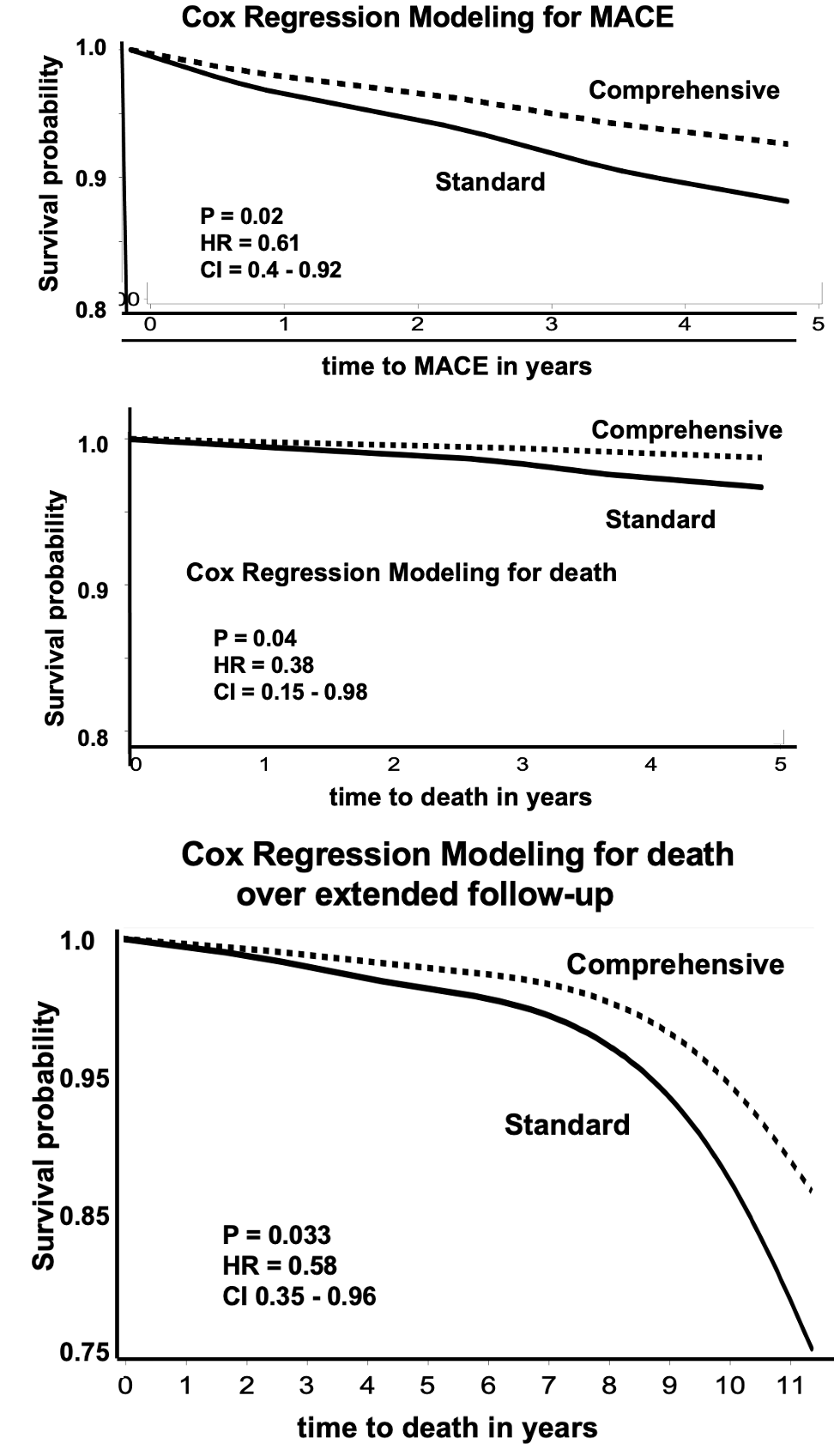


Supplement Figure S4 Gender and Events. Thirty-two percent of participants were female equally in comprehensive- and standard-care groups (table 1). For all participants in both groups, females had substantially less deaths, death or MI, and revascularisations than males (Supp) with significant differences between groups for cumulative death or MI, and revascularisations.


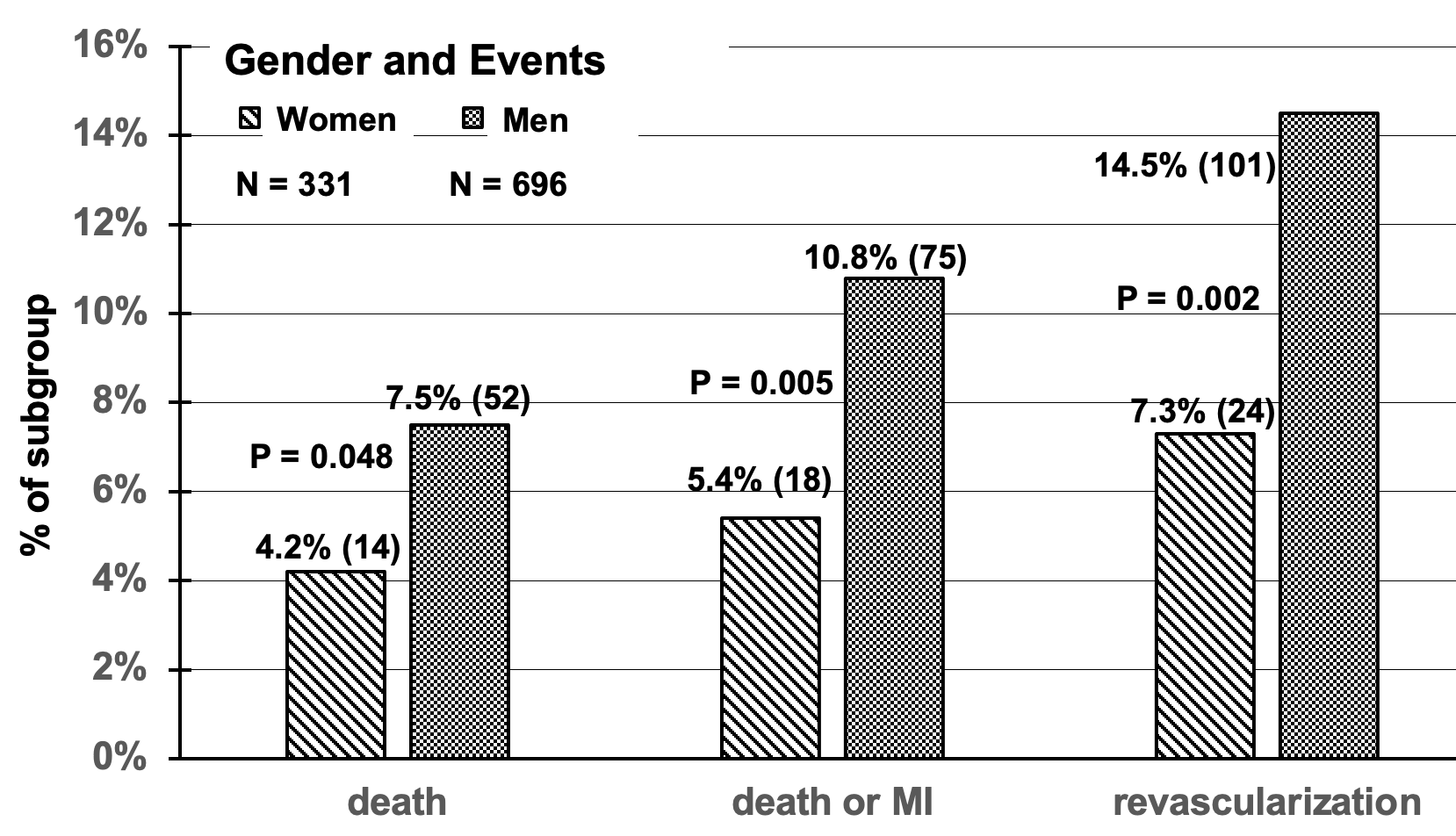

Supplement: ehaf356_Supplementary_Data [file ehaf356_supplementary_data.docx]
